# Supplementary material for: Research priorities for children’s cancer: a James Lind Alliance Priority Setting Partnership in the UK
Source: BMJ Open. 2023 Dec 20;13(12):e077387. doi: 10.1136/bmjopen-2023-077387 (PMC11148658; doi:10.1136/bmjopen-2023-077387)
Supplement: Supplementary data [file bmjopen-2023-077387supp001.pdf]

Supplementary material 1 Participant details first survey

	Response	Survivors (number=49)	Parents/relatives/friends/ (number=291)	Professionals (number=148)
**Gender**	Female	41 (84%)	260 (89%)	133 (90%)
	Male	8 (16%)	30 (10%)	14 (9%)
	Use another term	0 (0%)	0 (0%)	0 (0%)
	Prefer not to answer	0 (0%)	1 (0.3%)	1 (1%)
**Trans**	No	48 (98%)	281 (97%)	139 (94%)
	Yes	0 (0%)	0 (0%)	1 (1%)
	Prefer not to answer	0 (0%)	1 (0.3%)	0 (0%)
	Missing data	1 (2%)	9 (3%)	8 (5%)
**Ethnic group**	White	46 (94%)	276 (95%)	135 (91%)
	Asian or Asian British	1 (2%)	4 (1%)	6 (4%)
	Black African, Black Caribbean or Black British	0 (0%)	2 (1%)	2 (1%)
	Mixed/multiple ethnic groups	0 (0%)	4 (1%)	1 (1%)
	Other	2 (4%)	1 (0.3%)	2 (1%)
	Prefer not to answer	0 (0%)	2 (1%)	0 (0%)
	Missing data	0 (0%)	2 (1%)	2 (1%)
**Age (years)**	16-18	6 (12%)	0 (0%)	n/a
	19-24	4 (8%)	1 (0.3%)	3 (2%)
	25-34	18 (37%)	46 (16%)	33 (22%)
	35-44	12 (24%)	127 (44%)	39 (26%)
	45-54	7 (14%)	83 (29%)	46 (31%)
	55-64	1 (2%)	23 (8%)	23 (15%)
	65+	1 (2%)	5 (2%)	1 (1%)
	Prefer not to answer	0 (0%)	1 (0.3%)	1 (1%)
	Missing data	0 (0%)	5 (2%)	2 (1%)
**Country of residence (survivors/parents/relatives/friends) Country of work (professionals)**	England	36 (73%)	241 (83%)	123 (83%)
	Scotland	2 (4%)	12 (4%)	7 (5%)

	Wales	0 (0%)	13 (4%)	6 (4%)
	Northern Ireland	0 (0%)	4 (1%)	3 (2%)
	Other	10 (20%)	20 (7%)	8 (5%)
	Prefer not to answer	1 (2%)	1 (0.3%)	0 (0%)
	Missing data	0 (0%)	0 (0%)	1 (1%)
**Cancer first diagnosed with**	Bone tumour	6 (12%)	7 (2%)	n/a
	Brain or spinal tumour	3 (6%)	35 (12%)	n/a
	Germ cell tumour	0 (0%)	5 (2%)	n/a
	Kidney tumour	2 (4%)	18 (6%)	n/a
	Langerhans Cell Histiocytosis (LCH)	0 (0%)	5 (2%)	n/a
	Leukaemia	20 (41%)	132 (45%)	n/a
	Liver tumour	0 (0%)	1 (0.3%)	n/a
	Lymphoma	8 (16%)	19 (7%)	n/a
	Neuroblastoma	1 (2%)	22 (8%)	n/a
	Retinoblastoma	4 (8%)	14 (5%)	n/a
	Soft tissue sarcoma	4 (8%)	21 (7%)	n/a
	More than one cancer diagnosis	0 (0%)	5 (2%)	n/a
	Not sure	1 (2%)	2 (1%)	n/a
	Other	0 (0%)	5 (2%)	n/a
	Prefer not to answer	0 (0%)	0 (0%)	n/a
**Current situation**	On treatment	1 (2%)	90 (31%)	n/a
	Finished treatment in the last 0 to 12 months	0 (0%)	48 (16%)	n/a
	Finished treatment 1 to 5 years ago	3 (6%)	53 (18%)	n/a
	Finished treatment more than 5 years ago	40 (82%)	29 (10%)	n/a
	On treatment for relapse	2 (4%)	22 (8%)	n/a
	Receiving palliative care	0 (0%)	1 (0.3%)	n/a
	Passed away	n/a	45 (15%)	n/a
	Not sure	1 (2%)	0 (0%)	n/a
	Other	0 (0%)	3 (1%)	n/a
	Prefer not to answer	0 (0%)	0 (0%)	n/a
	Missing data	2 (4%)	0 (0%)	n/a

**Age at diagnosis**	Under 1	2 (4%)	24 (8%)	n/a
	1-3 years	9 (18%)	96 (33%)	n/a
	4-6 years	7 (14%)	66 (23%)	n/a
	7-9 years	9 (18%)	39 (13%)	n/a
	10-12 years	10 (20%)	26 (9%)	n/a
	13-15 years	11 (22%)	26 (9%)	n/a
	Over 16	0 (0%)	5 (2%)	n/a
	Not sure	0 (0%)	2 (1%)	n/a
	Prefer not to answer	0 (0%)	1 (0.3%)	n/a
	Missing data	1 (2%)	6 (2%)	n/a
**Professional group**	Allied health professional	n/a	n/a	49 (33%)
	Nurse	n/a	n/a	45 (30%)
	Doctor	n/a	n/a	27 (18%)
	Education professional	n/a	n/a	17 (11%)
	Social care professional	n/a	n/a	10 (7%)
