# Supplementary material for: Research priorities for children’s cancer: a James Lind Alliance Priority Setting Partnership in the UK
Source: BMJ Open. 2023 Dec 20;13(12):e077387. doi: 10.1136/bmjopen-2023-077387 (PMC11148658; doi:10.1136/bmjopen-2023-077387)
Supplement: Supplementary data [file bmjopen-2023-077387supp002.pdf]

Supplementary material 2 Participant details children and young people's surveys

	Response	Children and young people with cancer (n=61)	Siblings (n=10)
**Gender**	Male	22 (36%)	5 (50%)
	Female	38 (62%)	5 (50%)
	Prefer not to answer	1 (2%)	0 (0%)
**Age**	3-6 years	13 (21%)	1 (10%)
	7-9 years	17 (28%)	2 (20%)
	10-12 years	9 (15%)	2 (20%)
	13-15 years	16 (26%)	3 (30%)
	16-21 years	5 (8%)	1 (10%)
	Prefer not to answer	1 (2%)	1 (10%)
**Country of residence**	England	42 (69%)	6 (60%)
	Scotland	9 (15%)	2 (20%)
	Wales	6 (10%)	2 (20%)
	Northern Ireland	1 (2%)	0 (0%)
	Other	2 (3%)	0 (0%)
	Prefer not to answer	1 (2%)	0 (0%)
**Diagnosis**	Leukaemia	26 (43%)	3 (30%)
	Kidney tumour	7 (11%)	0 (0%)
	Lymphoma	7 (11%)	1 (10%)
	Brain/spinal tumour	5 (8%)	2 (20%)
	Soft tissue sarcoma	4 (7%)	0 (0%)
	Neuroblastoma	3 (5%)	2 (2%)
	Retinoblastoma	2 (3%)	0 (0%)
	Bone tumour	1 (2%)	0 (0%)
	More than one cancer diagnosis	1 (2%)	0 (0%)
	Other	2 (3%)	1 (10%)
	Prefer not to answer	2 (3%)	0 (0%)
	Do not know	1 (2%)	1 (10%)
**Ethnic group* (Children and young people with cancer n=36; Siblings n=7)**	White	31 (86%)	7 (100%)
	Asian or Asian British	1 (3%)	0 (0%)
	Black African, Black Caribbean or Black British	1 (3%)	0 (0%)
	Mixed/multiple ethnic groups	1 (3%)	0 (0%)
	Prefer not to answer	2 (6%)	0 (0%)
**Current situation* (Children and young people with cancer n=36; Siblings n=7)**	On treatment	12 (33%)	3 (43%)
	Finished treatment	23 (64%)	4 (57%)
	Other	1 (3%)	0 (0%)

*not asked in 4-7 year olds survey
