# Supplementary material for: Research priorities for children’s cancer: a James Lind Alliance Priority Setting Partnership in the UK
Source: BMJ Open. 2023 Dec 20;13(12):e077387. doi: 10.1136/bmjopen-2023-077387 (PMC11148658; doi:10.1136/bmjopen-2023-077387)
Supplement: Supplementary data [file bmjopen-2023-077387supp003.pdf]

Supplementary material 3 Participant details shortlisting survey

	Response	Survivors (number=27)	Parents/relatives/friends/ (number=210)	Professionals (number=90)
**Gender**	Female	23 (85%)	186 (89%)	75 (83%)
	Male	3 (11%)	21 (10%)	14 (16%)
	Use another term	1 (4%)	2 (1%)	0 (0%)
	Prefer not to answer	0 (0%)	0 (0%)	1 (1%)
	Missing data	0 (0%)	1 (0.5%)	0 (0%)
**Trans**	No	26 (96%)	206 (98%)	87 (97%)
	Yes	1 (4%)	0 (0%)	1 (1%)
	Prefer not to answer	0 (0%)	2 (1%)	2 (2%)
	Missing data	0 (0%)	2 (1%)	0 (5%)
**Ethnic group**	White	24 (89%)	199 (95%)	79 (88%)
	Asian or Asian British	1 (4%)	3 (1%)	5 (6%)
	Black African, Black Caribbean or Black British	0 (0%)	0 (0%)	1 (1%)
	Mixed/multiple ethnic groups	1 (4%)	5 (2%)	4 (4%)
	Other	1 (4%)	1 (0.5%)	0 (0%)
	Prefer not to answer	0 (0%)	1 (0.5%)	1 (1%)
	Missing data	0 (0%)	1 (0.5%)	0 (0%)
**Age (years)**	16-18	5 (19%)	1 (0.5%)	0 (0%)
	19-24	9 (33%)	3 (1%)	0 (0%)
	25-34	6 (22%)	24 (11%)	23 (26%)
	35-44	3 (11%)	98 (47%)	26 (29%)
	45-54	3 (11%)	57 (27%)	30 (33%)
	55-64	1 (4%)	19 (9%)	8 (9%)
	65+	0 (0%)	7 (3%)	1 (1%)
	Prefer not to answer	0 (0%)	0 (0%)	2 (2%)
	Missing data	0 (0%)	1 (0.5%)	0 (0%)
**Country of residence (survivors/parents/relatives/friends) Country of work (professionals)**	England	25 (93%)	170 (81%)	78 (87%)

	Scotland	0 (0%)	17 (8%)	6 (7%)
	Wales	0 (0%)	10 (5%)	3 (3%)
	Northern Ireland	0 (0%)	2 (1%)	1 (1%)
	Other	1 (4%)	10 (5%)	1 (1%)
	Prefer not to answer	1 (4%)	1 (0.5%)	1 (1%)
	Missing data	0 (0%)	0 (0%)	0 (0%)
**Cancer first diagnosed with**	Bone tumour	4 (15%)	6 (3%)	n/a
	Brain or spinal tumour	2 (7%)	26 (12%)	n/a
	Germ cell tumour	0 (0%)	3 (1%)	n/a
	Kidney tumour	0 (0%)	10 (5%)	n/a
	Langerhans Cell Histiocytosis (LCH)	0 (0%)	0 (0%)	n/a
	Leukaemia	10 (37%)	113 (54%)	n/a
	Liver tumour	0 (0%)	0 (0%)	n/a
	Lymphoma	4 (15%)	15 (7%)	n/a
	Neuroblastoma	1 (4%)	9 (4%)	n/a
	Retinoblastoma	1 (4%)	8 (4%)	n/a
	Soft tissue sarcoma	3 (11%)	14 (7%)	n/a
	More than one cancer diagnosis	1 (4%)	1 (0.5%)	n/a
	Not sure	0 (0%)	0 (0%)	n/a
	Other	1 (4%)	4 (2%)	n/a
	Prefer not to answer	0 (0%)	0 (0%)	n/a
	Missing data	0 (0%)	1 (0.5%)	n/a
**Current situation**	On treatment	2 (7%)	58 (28%)	n/a
	Finished treatment in the last 0 to 12 months	1 (4%)	21 (10%)	n/a
	Finished treatment 1 to 5 years ago	5 (19%)	60 (29%)	n/a
	Finished treatment more than 5 years ago	19 (70%)	30 (14%)	n/a
	On treatment for relapse	0 (0%)	8 (4%)	n/a
	Receiving palliative care	0 (0%)	2 (1%)	n/a
	Passed away	n/a	26 (12%)	n/a
	Not sure	0 (0%)	0 (0%)	n/a
	Other	0 (0%)	4 (2%)	n/a

	Prefer not to answer	0 (0%)	0 (0%)	n/a
	Missing data	0 (0%)	1 (0.5%)	n/a
**Age at diagnosis**	Under 1	1 (4%)	16 (8%)	n/a
	1-3 years	1 (4%)	63 (30%)	n/a
	4-6 years	6 (22%)	54 (26%)	n/a
	7-9 years	4 (15%)	25 (12%)	n/a
	10-12 years	3 (11%)	26 (12%)	n/a
	13-15 years	10 (37%)	23 (11%)	n/a
	Over 16	2 (7%)	1 (0.5%)	n/a
	Not sure	0 (0%)	0 (0%)	n/a
	Prefer not to answer	0 (0%)	0 (0%)	n/a
	Missing data	0 (0%)	2 (1%)	n/a
**Professional group**	Allied health professional	n/a	n/a	25 (28%)
	Nurse	n/a	n/a	30 (33%)
	Doctor	n/a	n/a	25 (28%)
	Education professional	n/a	n/a	4 (4%)
	Social care professional	n/a	n/a	4 (4%)
	Other	n/a	n/a	2 (2%)
