# Supplementary material for: Research priorities for children’s cancer: a James Lind Alliance Priority Setting Partnership in the UK
Source: BMJ Open. 2023 Dec 20;13(12):e077387. doi: 10.1136/bmjopen-2023-077387 (PMC11148658; doi:10.1136/bmjopen-2023-077387)
Supplement: Author's manuscript [file bmjopen-2023-077387.draft_revisions.pdf]

BMJ Open is committed to open peer review. As part of this commitment we make the peer review history of every article we publish publicly available.

When an article is published we post the peer reviewers' comments and the authors' responses online. We also post the versions of the paper that were used during peer review. These are the versions that the peer review comments apply to.

The versions of the paper that follow are the versions that were submitted during the peer review process. They are not the versions of record or the final published versions. They should not be cited or distributed as the published version of this manuscript.

BMJ Open is an open access journal and the full, final, typeset and author-corrected version of record of the manuscript is available on our site with no access controls, subscription charges or pay-per-view fees (http://bmjopen.bmj.com).

If you have any questions on BMJ Open's open peer review process please email info.bmjopen@bmj.com

BMJ Open

Research priorities for children's cancer: a James Lind Alliance priority setting partnership in the United Kingdom

Journal:	*BMJ Open*
Manuscript ID	bmjopen-2023-077387
Article Type:	Original research
Date Submitted by the Author:	03-Jul-2023

Complete List of Authors:	Aldiss, Susie; University of Surrey, School of Health Sciences<br>Hollis, Rachel; Leeds Children's Hospital<br>Phillips, Bob; Leeds Teaching Hospitals NHS Trust, Department of Paediatric Haematology and Oncology; University of York, Hull-York Medical School and Centre for Reviews and Dissemination<br>Ball-Gamble, Ashley; Children's Cancer and Leukaemia Group,<br>Brownsdon, Alex; Patient Representative on the Children's Cancer Priority Setting Partnership steering group<br>Chisholm, Julia; The Royal Marsden NHS Foundation Trust; Institute of Cancer Research Sutton<br>Crowther, Scott; Parent Representative on the Children's Cancer Priority Setting Partnership steering group<br>Dommett, Rachel; Bristol Royal Hospital for Children<br>Gower, Jonathan; James Lind Alliance, National Institute for Health Research Evaluation, Trials and Studies Coordinating Centre<br>Hall, Nigel; Southampton Children's Hospital; University of Southampton Faculty of Medicine, University Surgery Unit<br>Hartley, Helen; Alder Hey Children's NHS Foundation Trust<br>Hatton, Jenni; University Hospitals Birmingham NHS Foundation Trust<br>Henry, Louise; The Royal Marsden NHS Foundation Trust<br>Langton, Loveday; Parent Representative on the Children's Cancer Priority Setting Partnership steering group<br>Maddock, Kirsty; Leeds Children's Hospital<br>Malik, Sonia; Young Lives Vs Cancer<br>McEvoy, Keeley; Leeds Children's Hospital, Medical Needs Teaching Service<br>Morgan, Jessica; Leeds Teaching Hospitals NHS Trust, Department of Paediatric Haematology and Oncology; University of York, Centre for Reviews and Dissemination<br>Morris, Helen; University Hospitals Bristol NHS Foundation Trust<br>Parke, Simon; Royal Devon and Exeter NHS Foundation Trust<br>Picton, Sue; Leeds Children's Hospital<br>Reed-Berendt, Rosa; Great Ormond Street Hospital for Children NHS Foundation Trust, Psychological Services<br>Saunders, Dan; The Christie NHS Foundation Trust<br>Stewart, Andy; Parent Representative on the Children's Cancer Priority Setting Partnership Steering Group<br>Tarplee-Morris, Wendy; The Little Princess Trust<br>Walsh, Amy; Parent Representative on the Children's Cancer Priority Setting Partnership Steering Group<br>Watkins, Anna; Parent Representative on the Children's Cancer Priority

	Setting Partnership steering group<br>Weller, David; The University of Edinburgh Edinburgh Medical School<br>Gibson, Faith; University of Surrey, School of Health Sciences; Great Ormond Street Hospital For Children NHS Foundation Trust, ORCHID Centre for Outcomes and Experience Research in Child Health, Illness and Disability
Keywords:	Paediatric oncology < PAEDIATRICS, Patient Participation, Surveys and Questionnaires, Paediatric oncology < ONCOLOGY

SCHOLARONE™ Manuscripts

I, the Submitting Author has the right to grant and does grant on behalf of all authors of the Work (as defined in the below author licence), an exclusive licence and/or a non-exclusive licence for contributions from authors who are: i) UK Crown employees; ii) where BMJ has agreed a CC-BY licence shall apply, and/or iii) in accordance with the terms applicable for US Federal Government officers or employees acting as part of their official duties; on a worldwide, perpetual, irrevocable, royalty-free basis to BMJ Publishing Group Ltd ("BMJ") its licensees and where the relevant Journal is co-owned by BMJ to the co-owners of the Journal, to publish the Work in this journal and any other BMJ products and to exploit all rights, as set out in our licence.

The Submitting Author accepts and understands that any supply made under these terms is made by BMJ to the Submitting Author unless you are acting as an employee on behalf of your employer or a postgraduate student of an affiliated institution which is paying any applicable article publishing charge ("APC") for Open Access articles. Where the Submitting Author wishes to make the Work available on an Open Access basis (and intends to pay the relevant APC), the terms of reuse of such Open Access shall be governed by a Creative Commons licence – details of these licences and which Creative Commons licence will apply to this Work are set out in our licence referred to above.

Other than as permitted in any relevant BMJ Author's Self Archiving Policies, I confirm this Work has not been accepted for publication elsewhere, is not being considered for publication elsewhere and does not duplicate material already published. I confirm all authors consent to publication of this Work and authorise the granting of this licence.

For peer review only

Research priorities for children's cancer: a James Lind Alliance priority setting partnership in the United Kingdom

Susie Aldiss, Rachel Hollis, Bob Phillips, Ashley Ball-Gamble, Alex Brownsdon, Julia Chisholm, Scott Crowther, Rachel Dommett, Jonathan Gower, Nigel Hall, Helen Hartley, Jenni Hatton, Louise Henry, Loveday Langton, Kirsty Maddock, Sonia Malik, Keeley McEvoy, Jess Morgan, Helen Morris, Simon Parke, Sue Picton, Rosa Reed-Berendt, Dan Saunders, Andy Stewart, Wendy Tarplee-Morris, Amy Walsh, Anna Watkins, David Weller, Faith Gibson

Susie Aldiss, Research Fellow, School of Health Sciences, University of Surrey, Guildford, UK.

Rachel Hollis, PSP Lead, Honorary Nurse Advisor, Leeds Children's Hospital, Leeds, UK

Bob Phillips, Senior Lecturer and Consultant in Paediatric Oncology, Department of Paediatric Haematology and Oncology, Leeds Teaching Hospitals NHS Trust, Leeds and Hull-York Medical School and Centre for Reviews and Dissemination, University of York, York, UK

Ashley Ball-Gamble, Chief Executive Officer, Children's Cancer and Leukaemia Group, Leicester, UK

Alex Brownsdon, Patient Representative on the Children's Cancer Priority Setting Partnership steering group, London, UK

Julia Chisholm, Consultant in Paediatric and Adolescent Oncologist, The Royal Marsden NHS Foundation Trust and Institute of Cancer Research, Sutton, UK

Scott Crowther, Parent Representative on the Children's Cancer Priority Setting Partnership steering group, Coventry, UK

Rachel Dommett, Consultant in Paediatric Leukaemia and Aftercare, Bristol Royal Hospital for Children, Bristol, UK

Jonathan Gower, James Lind Alliance Advisor, The James Lind Alliance, National Institute for Health Research Evaluation, Trials and Studies Coordinating Centre, University of Southampton, Southampton, UK

Nigel Hall, Consultant Paediatric Surgeon, Southampton Children's Hospital; Associate Professor of Paediatric Surgery, University of Southampton, Southampton, UK

Helen Hartley, Specialist Paediatric Physiotherapist - Acute Neurosciences/ Oncology, Alder Hey Children's NHS Foundation Trust, Liverpool, UK

Jenni Hatton, Advanced Pharmacist Clinical Oncology, University Hospitals Birmingham NHS Foundation Trust, Birmingham, UK

Louise Henry, Senior Specialist Dietitian (Paediatrics and TYA), The Royal Marsden NHS Foundation Trust, Sutton, UK

Loveday Langton, Parent Representative on the Children's Cancer Priority Setting Partnership steering group, London, UK

Kirsty Maddock, Speech & Language Therapist, Leeds Children's Hospital, Leeds, UK

Sonia Malik, Head of Policy and Influencing, Young Lives vs Cancer, London, UK

For peer review only - http://bmjopen.bmj.com/site/about/guidelines.xhtml

Keeley McEvoy, Assistant Headteacher, Medical Needs Teaching Service, Leeds Children's Hospital, Leeds, UK

Jess Morgan, Senior Research Fellow in Paediatric Oncology, Department of Paediatric Haematology and Oncology, Leeds Teaching Hospitals NHS Trust, Leeds and Centre for Reviews and Dissemination, University of York, York, UK

Helen Morris, Children's Cancer Network Lead Nurse, Children, Teenage and Young Adult Cancer Operational Delivery Network, South West, Bristol, UK

Simon Parke, Consultant Paediatrician, Royal Devon and Exeter NHS Foundation Trust, UK

Sue Picton, Consultant Paediatric and Adolescent Oncologist, Leeds Children's' Hospital/ Martin House Hospice, UK

Rosa Reed-Berendt, Clinical Psychologist, Psychological Services, Great Ormond Street Hospital for Children NHS Foundation Trust, London, UK.

Dan Saunders, Consultant in Clinical Oncology, The Christie NHS Foundation Trust, Manchester, UK

Andy Stewart, Parent Representative on the Children's Cancer Priority Setting Partnership Steering Group, Perth, UK

Wendy Tarplee-Morris, Co-founder and Director of Service and Impact, The Little Princess Trust, Hereford, UK

Amy Walsh, Parent Representative on the Children's Cancer Priority Setting Partnership Steering Group, Keswick, UK

Anna Watkins, Parent Representative on the Children's Cancer Priority Setting Partnership Steering Group, London, UK

David Weller, Professor of General Practice, University of Edinburgh, Edinburgh, UK

Faith Gibson, ORCHID Centre for Outcomes and Experience Research in Child Health, Illness and Disability, Great Ormond Street Hospital for Children NHS Foundation Trust, London, UK and School of Health Sciences, University of Surrey, Guildford, UK.

Corresponding author:

Susie Aldiss, School of Health Sciences, Kate Granger Building, 30 Priestley Road, Surrey Research Park, Guildford, GU2 7YH, UK. s.aldiss@surrey.ac.uk

Keywords: Cancer, children, research priorities, James Lind Alliance, patient and public involvement

For peer review only - http://bmjopen.bmj.com/site/about/guidelines.xhtml
For peer review only

ABSTRACT

Objectives

To engage children who have experienced cancer, childhood cancer survivors, their families, and professionals to systematically identify and prioritise research questions about childhood cancer to inform the future research agenda.

Design

James Lind Alliance Priority Setting Partnership.

Setting

UK health service and community.

Methods

A steering group oversaw the initiative. Potential research questions were collected in an online survey, then checked to ensure they were unanswered. Shortlisting via a second online survey identified the highest priority questions. A parallel process with children was undertaken. A final consensus workshop was held to determine the Top 10 priorities.

Participants

Children and survivors of childhood cancer, diagnosed before age 16, their families, friends, and professionals who work with this population.

Results

Four hundred and eighty-eight people submitted 1299 potential questions. These were refined into 108 unique questions; four were already answered and three were under active study, therefore removed. Three hundred and twenty-seven respondents completed the shortlisting survey. Seventy-one children submitted questions in the children's surveys, eight children attended a workshop to prioritise these questions. The Top 5 questions from children were taken to the final workshop where 23 questions in total were discussed by 25 participants (young adults, carers and professionals). The top priority was, 'Can we find effective and kinder (less burdensome, more tolerable, with fewer short- and long-term effects) treatments for children with cancer, including relapsed cancer?'

Conclusions

We have identified research priorities for children's cancer from the perspectives of children, survivors, their families, and the professionals who care for them. Questions reflect the breadth of the cancer experience, including diagnosis, relapse, hospital experience, support during/after treatment and the long-term impact of cancer. These should inform funding of future research as they are the questions that matter most to the people who could benefit from research.

For peer review only - http://bmjopen.bmj.com/site/about/guidelines.xhtml

ARTICLE SUMMARY

Strengths and limitations of this study

- We made use of the well-established and transparent James Lind Alliance methodology and clearly describe the process and decision making which led to the final Top 10 research priorities;
- The process followed ensures that these priorities came directly from those who are the most affected by childhood cancer but rarely influence the research agenda;
- We ensured the priorities of patients/survivors, parents/relatives/friends and professionals were given equal weighting at the interim priority setting stage;
- We used innovative methods to hear directly from children about their priorities for future research through surveys and a workshop specifically designed for them;
- Underrepresented groups in the survey submissions included people from minority ethnic groups, males and primary health care professionals.

Word count = 4396

For peer review only

INTRODUCTION

Annually there are around 1,800 new cases of cancer in children in the UK.[1] While research over the last four decades has dramatically increased the overall five-year survival rate for all childhood cancers to around 84%[2] further research is needed to not only improve outcomes for all types of cancer, but to support all children to live long, healthy and happy lives.

Historically, topics of healthcare research in children's cancer have been driven by perspectives of researchers and the pharmaceutical industry, meaning what is most important to children, survivors, their families and the professionals who care for them, has sometimes been overlooked. Prioritising areas for research as identified by children and carers is crucial. There is increasing evidence that research questions and outcomes prioritised by professionals may not be aligned to those experiencing the disease.[3] Patients and carers tend to prioritise non-drug treatment research while ongoing research strategies are dominated by drug evaluations.[4] This mismatch in priorities is particularly relevant for children due to their unique physiological and psychosocial status and relative rarity of cancer. Increasingly, research funders are asking if proposed research is a priority for patients.

The James Lind Alliance (JLA) is a non-profit making initiative bringing together patients, carers and professionals in Priority Setting Partnerships (PSPs) focusing on specific health conditions http://www.jla.nihr.ac.uk/priority-setting-partnerships/). JLA PSPs identify and prioritise unanswered questions, so researchers and research funders are aware of the issues that matter most to those who could benefit from that research.[5]

In 2019, Children's Cancer and Leukaemia Group (CCLG; https://www.cclg.org.uk/) and The Little Princess Trust (https://www.littleprincesses.org.uk/) partnered with the JLA on the Children's Cancer PSP. One of our primary goals was to prioritise the voice of children about what research should be undertaken. Previous PSPs have sought to involve children and young people, but in the final reporting it is evident that few children, especially young children, had been engaged through the process.[6] We recognised the challenges of engaging with these populations, in terms of reach and accessibility of information, and determined we would invest time and resources, in exploring and resolving any challenges that could impact on participation.

Following the JLA methodology, we aimed to conduct a UK-wide research prioritisation exercise for childhood cancer to inform decisions of research funders and support the case for research in this underserved group.[7]

METHODS

Methodology followed the JLA process[5] the protocol is available from: https://www.jla.nihr.ac.uk/priority-setting-partnerships/childrens-cancer/.

Set-up

Project management

There was a coordinating team of four researchers, nurses and clinicians. An expert steering group (all co-authors) oversaw the project, approved aims/objectives, survey materials, contributed to data analysis and summary question formation, and provided expert opinions for evidence checking. The steering group included parents of a child with cancer (n=5); an adult survivor of childhood cancer; a range of professionals reflecting the multidisciplinary nature of the care of children with cancer including: a teacher, General Practitioner (GP), surgeon, pharmacist, dietitian, speech and

For peer review only - http://bmjopen.bmj.com/site/about/guidelines.xhtml

language therapist, clinical psychologist, physiotherapist, nurses (n=2), doctors (n=6) and representatives from the third sector (n=3), including the charities funding the project. The JLA chair (JG) provided neutral facilitation of meetings. The steering group identified potential partners, mainly children's cancer charities and professional networks, who were approached to assist with survey dissemination.

Scope

This project focused on cancer and cancer-like conditions in children aged 0 to <16 at initial diagnosis. The scope, kept intentionally broad, included questions on any aspect of the cancer experience (Figure 1).

Our aim was, *'To identify gaps and unanswered questions in research about children's cancer from patients, carers and professionals' perspectives and then prioritise those that these groups agree are the most important for research to address.'*

Process

Figure 2 summarises the complete process.

Stage 1a – Gathering questions – initial survey

The survey was developed by the steering group and built using Qualtrics software. It was piloted with eight adult survivors of childhood cancer, nine parents and two professionals outside the steering group and adapted to incorporate their feedback. The survey launched on 9/9/2020 and closed on 8/1/2021. The following groups were invited to participate:

- People diagnosed with cancer before their 16th birthday;
- Relatives/friends/partners/carers of someone diagnosed with cancer before their 16th birthday;
- Professionals involved in diagnosing or treating children who have cancer or had cancer under 16;
- Professionals involved in the care of children who have cancer or had cancer under 16 and/or their families.

Respondents could submit up to eight questions about any aspect of children's cancer they considered important and unanswered. Basic demographic data were requested, and a box was available for free-text comments. Partners promoted the survey through websites, social media, newsletters, and email.

Stage 1b – Gathering questions from children and young people

A subgroup of the steering group was established to focus on our engagement with children. This consisted of two researchers, a teacher, doctor, health play specialist, parent, clinical psychologist, and charity representative. Our initial intention had been to run a series of face-to-face workshops with children to collect questions, this was not possible due to the COVID pandemic until the final workshop in the PSP process.

We determined that the best way to reach children would be through their parents/carers. Three survey versions were built using Qualtrics software, aimed at children of different ages (4-7, 8-12, 13-15 years). Surveys were piloted with children and young people. They varied in complexity of language used in the introduction and questions, and surveys for young people contained more questions seeking demographic information: participants could complete whichever survey version

For peer review only - http://bmjopen.bmj.com/site/about/guidelines.xhtml

they preferred. Animations were developed to assist parents explain the project and survey to their child(ren) (surveys/animations available here: https://www.jla.nihr.ac.uk/priority-setting-partnerships/childrens-cancer/).

Surveys were launched on 6/9/2021 and closed on 16/11/2021 inviting participants who:

- were diagnosed with cancer before their 16th birthday;
- have a brother or sister with cancer now or who had cancer when they were younger;
- have a friend with cancer now or who had cancer when they were younger.

Respondents were invited to submit up to eight questions/topics about any aspect of children's cancer they considered important. Surveys were promoted through the PSP's Partners, social media, and posters were sent to all UK Principal Treatment Centres.

Stage 2a - Refining questions from the initial survey

Submitted questions were examined in detail and free-text sections studied for further questions.

Organising the questions

Initial coding was carried out by coordinating team members (SA, FG). Questions were grouped into themes. During coding, potential 'out-of-scope' questions were identified (see Box 1 for criteria used). Identification of out-of-scope questions was an iterative process, checked and agreed by the steering group.

Similar questions were grouped to form summary questions. The aim was to retain the sense of what respondents meant, but in the form of a clear question. Steering group members met online in small groups to review summary questions within their area of expertise/experience, to confirm the grouping of questions, and wording of each summary question. The steering group reviewed the whole summary question list.

Box 1 Out-of-scope question categories and examples

1. The question was ambiguous, was interpreted in different ways by steering group members and the meaning could not be resolved following discussion:
   'Remaining scar tissue'
   'How research is going'

2. Questions not answerable by research:
   'Why does paediatric cancer research receive so very little funding?'
   'Who is present when you give the diagnosis'.

3. Questions submitted by people whose experience was not of childhood cancer as defined by our project scope - there were a few parent respondents whose child was over 16 at diagnosis. These questions were checked to verify that all the themes within them had been covered by 'in scope' questions.

Evidence searching

Searches were undertaken to identify questions answered by existing evidence. A search strategy was agreed with the steering group (see question verification form: https://www.jla.nihr.ac.uk/priority-setting-partnerships/childrens-cancer/). Searches were carried

For peer review only - http://bmjopen.bmj.com/site/about/guidelines.xhtml

out by SA in January-May 2022. Searches were limited to evidence published in the last five years (since January 2017) and focused on evidence gathered from multiple studies (e.g. systematic reviews, qualitative meta-synthesis). Searches were undertaken for ongoing studies which included personal communication with experts in the field and steering group members' knowledge of current research.

Stage 2b – Refining questions from children and young people

The same process was followed for refining questions from the children and young people's surveys. Questions were grouped into themes by SA with support from FG, similar questions were merged, and summary questions created. Out-of-scope questions were removed, if they were unrelated to cancer or were unclear (e.g. 'cost to hospital', 'wildlife'). The subgroup met online to review summary questions and out-of-scope questions, with further checking undertaken via email until agreement was reached.

Stage 3 – Question prioritisation

Shortlisting survey preparation

The steering group discussed whether to take all unanswered questions to the shortlisting survey or shorten the list to make the survey quicker to complete. The group chose not to remove any questions.

To ensure questions were easy to understand, they were reviewed by patient and parent members of the steering group and a health information specialist from one of the funding charities. Questions were simplified following this review and definitions of words added.

Shortlisting survey

The shortlisting survey was created using Qualtrics software, launched 3/8/2022 and closed 30/9/2022. Invitations mirrored the initial survey, and it was publicised using the same methods. Initial survey participants who left contact details were emailed directly.

To shorten the question list, respondents were invited to read the 101 questions and select those that were most important to them. Questions selected were added to their own personal 'shortlist' ready for them to make their final selection of up to 15 questions.

Questions were grouped into:

1. Side-effects and management
2. Treatment
3. Education
4. Physical activity, play and therapies
5. Long-term effects and follow-up care
6. Communication and information sharing
7. Psychological and social wellbeing
8. Food and nutrition
9. Healthcare delivery
10. Causes of cancer, diagnosis and research

Survey fatigue was minimised by randomisation of section order and questions.

Results were analysed in three groups: 1) patients/survivors, 2) parents/friends/relatives, 3) professionals. This gave equal weight to each group's choices as more parents/friends/relatives took

For peer review only

For peer review only - http://bmjopen.bmj.com/site/about/guidelines.xhtml

part. Questions were given a rank depending on number of votes and ordered from highest to lowest for each group. The steering group reviewed and compared respondent groups and decided to take the Top 10 questions for each of the three groups to the workshop. This ensured that what was important to each group would be considered and resulted in 21 questions being shortlisted, as some questions were shared priorities.

Stage 4a – Workshop with children and young people

The children and young people's workshop took place in October 2022. The workshop was facilitated by SA and FG following the methodology used by the Juvenile Idiopathic Arthritis PSP.[8] Children were given a choice of seven envelopes, each containing questions on a different topic with a total of 31 questions. Topics were:

1. Family, friends, and pets
2. Treatments and medicines
3. Being poorly, side-effects and long-term effects
4. Being in hospital
5. Emotions, worries and getting help or support
6. School and education
7. Getting the information you need.

Each participant chose the topic which was most important to them. Envelopes were opened, and participants placed the questions on the table in groups of most, medium or least important. Participants were invited to add more questions if anything of importance to them was missing. They were given three stickers to vote for their Top 3 questions. Questions were placed in order of most to least votes and a discussion followed to agree the 'Top 5'; these were taken to the final workshop.

Stage 4b – Top 10 Prioritisation

The final prioritisation workshop took place in November 2022. Participants who left their contact details in the survey were invited to attend as were patient and parent representatives on the steering group. Steering group contacts were used to ensure participation from a broad range of professionals across the field.

Prior to the workshop, participants were asked to individually rank the questions in order of importance. The workshop was chaired by JG and supported by two JLA facilitators. Participants were split into three pre-allocated groups ensuring a balance of multi-disciplinary professionals, young adults, and parents/relatives. In each group, participants shared their three highest and lowest ranking questions. Participants were told which questions were in the children's Top 5.

During facilitated discussion, the groups ordered the questions from highest to lowest priority. The ranking from the three groups were combined. In a second session, groups were re-allocated and the combined ranking was discussed. Following this discussion, the group rankings were again collated, and all participants formed one group to debate and agree the Top 10.

Patient and Public Involvement

Parent and patient representatives were involved as equal members of the steering group and in all stages of the prioritisation process. Patients and carers were survey respondents. Children were included in a parallel process. Young adults and parents/relatives attended the final prioritisation

For peer review only - http://bmjopen.bmj.com/site/about/guidelines.xhtml

workshop alongside professionals as equal stakeholders. Participants were reimbursed for travel/overnight accommodation costs.

RESULTS

Figure 2 provides an overview of the number of respondents at each stage.

Initial survey

Four-hundred and eighty-eight people submitted 1299 questions. Respondents included 49 (10%) patients/survivors, 291 (60%) parents/relatives/friends and 148 (30%) professionals. Most parents/relative/friends were parents (n=271; 93%), 15 (5%) were relatives and five (2%) friends. Supplementary material 1 shows respondent demographics.

One-hundred and thirty-nine out-of-scope questions were removed; Box 1 illustrates examples. Following the combining of similar questions and rewording to form summary questions, 108 questions remained.

Analysis of uncertainties

Four questions were already answered, and three the focus of ongoing studies. For some questions, no reviews or ongoing studies were identified. If reviews only partly answered a question, these were recorded as unanswered. The steering group discussed all questions ensuring consensus agreement of answered/unanswered questions; 101 questions were unanswered.

Children and young people's surveys

Seventy-one respondents submitted 252 questions/topics. Sixty-one respondents were children and young people who had experienced cancer (aged 3-21) and ten were siblings (aged 4-19). No friends participated. See supplementary material 2 for demographics. For brevity, we refer to submissions as 'questions'; nearly all submissions were not written as questions. Thirteen questions were identified as out-of-scope and removed. Responses were summarised into 24 questions.

Shortlisting survey

Ratings were submitted by 327 respondents. Like the initial survey, the largest respondent group was parents/relatives/friends (64%, n=210; including 197 parents, 10 relatives, three friends), followed by professionals (28%, n=90) and patients/survivors (8%, n=27). See supplementary material 3 for demographics.

Children and young people's workshop

Eight children and young people aged 8-16 attended; three were siblings. Their diagnoses included lymphoma and leukaemia.

During discussion, seven additional questions were created about family, friends, and pets and six were added on topics that were important to participants. The Top 5 are shown in Table 1. Three of the questions were closely aligned to those already going to the final workshop from the shortlisting survey (priorities 2, 4 and 5). For priority 4, the children and young people's version of the question had an extra part about starting treatment in the right place, this version was taken to the final workshop. Priorities 1 and 3 from children and young people were new and were added into the list, making 23 questions in total for the final workshop.

For peer review only - http://bmjopen.bmj.com/site/about/guidelines.xhtml

For peer review only

Table 1 Children and young people's Top 5 and questions for the final workshop

Rank	Top 5 questions from the children and young people's workshop	Question going to the final workshop from the shortlisting survey
1	How can we make being in hospital a better experience for children and young people? (like having better food, internet, toys, and open visiting so other family members can be more involved in the child's care)	
2	How can we prevent cancer in children and young people?	Why do children develop cancer (including the role that genetics plays) and could it be prevented?
3	How can we make more accessible treatments that are closer to home, in shared care hospitals?	
4	How can we speed up the process of getting diagnosed and starting treatment in the right place?	How can time to diagnosis be improved for children with suspected cancer?
5	What are the best ways to help children and young people with their worries and make them feel happier?	What are the best ways to provide emotional support for children and their families 1) around the time of diagnosis, 2) during treatment and 3) after treatment (including survivors who are now adults)?

Final workshop

Twenty-five participants attended: four young adults who had experienced childhood cancer, five parents and one grandparent of a child who had cancer, and 15 professionals who work with this population. Professional roles varied and included nurses, doctors, a social worker, health play specialist, dietitian, clinical psychologist, physiotherapist and chaplain. One participant was a steering group member.

Top 10 Prioritisation strategies

Although the three groups worked independently, they all applied similar prioritisation strategies:

Ensuring children's views were represented

All groups wanted to ensure the Top 10 questions included most, if not all, questions from the children's Top 5. When the groups were told which questions were important to children, those question cards were picked out and moved up the ranking. Most of these questions remained in the Top 10, or just outside, for the duration of the discussions.

Opting for questions that could include other questions/overlap

Groups considered which questions overlapped and could cover other questions. For example, 'Can we find effective and kinder (less burdensome, more tolerable, with fewer short and long-term effects) treatments for children with cancer, including relapsed cancer?' mentions side-effects and

For peer review only

so could include, 'What are the best ways to reduce, predict and manage the side-effects of treatment for children (including life threatening side-effects)?

Opting for questions focussed on intervention rather than description

Groups were clear that although it is useful to describe a problem, it is action through intervention that is required to improve children's and families' experiences. Therefore, 'Are the psychological, practical, and financial support needs of children with cancer, survivors, and their families being met during treatment and beyond? How can access to this support be improved and what further support would they like?' was placed higher in the rankings than 'What is the psychological and social impact of cancer and treatment on children and their families during treatment and in the long-term; what factors affect these impacts?' as the latter question involves description, rather than action.

Opting for questions that could have wider impact

Initially, most participants selected their top three questions reflective of their personal experience or area they worked within. During discussions, their opinions changed, and groups decided that the Top 10 questions should be generic and have the potential to have the greatest impact on as many children and families as possible. For example, 'How can experiences of having a Hickman line be improved for children with cancer?' was considered too specific and did not apply to all children.

Ensuring all themes within the questions were represented

Groups tried to cluster questions into similar themes, such as support, treatment, care, side-effects, their aim being to include each 'theme' in the Top 10. For example, the question about relapse was moved up during discussions as this was not covered by any other question.

Group discussion and decision-making

From the outset, there were some questions that were high priority for many and stayed high in the Top 10 throughout the workshop. The question ranked as top priority, 'Can we find effective and kinder (less burdensome, more tolerable, with fewer short and long-term effects) treatments for children with cancer, including relapsed cancer?' was the top priority for all three groups after the first group discussion. After the second group discussion, all three groups had the same questions ranked one to five, which remained in the same positions in the final Top 10.

The final group discussion focussed on whether to include, 'What is the relationship between chronic fatigue syndrome, fibromyalgia, chronic pain and treatment for childhood cancer?' in the Top 10 (it was at number 11). This push for inclusion came from two young adults who said these long-term effects had a huge impact on their lives and had experienced a lack of recognition and support. There was a group vote and the decision was made to move this question up to number 10 and move, 'What are the best ways to provide emotional support for children and their families 1) around the time of diagnosis, 2) during treatment and 3) after treatment (including survivors who are now adults)?' down to number 11 as this was covered by the broader question, about support at number 3.

The final Top 10 priorities are shown in Box 2 alongside the other 13 questions discussed.

For peer review only - http://bmjopen.bmj.com/site/about/guidelines.xhtml

For peer review only

Box 2 Top 10 research priorities for Children's cancer and the additional 13 questions discussed at the workshop

1. Can we find effective and kinder (less burdensome, more tolerable, with fewer short and long-term effects) treatments for children with cancer, including relapsed cancer?
2. Why do children develop cancer (including the role that genetics plays) and could it be prevented?[1]
3. Are the psychological, practical, and financial support needs of children with cancer, survivors, and their families being met during treatment and beyond? How can access to this support be improved and what further support would they like?[2]
4. How can we speed up the process of getting diagnosed and starting treatment in the right place?[1]
5. Why do children relapse, how can it be prevented, and what are the best ways to identify relapse earlier?
6. How can we make being in hospital a better experience for children and young people? (like having better food, internet, toys, and open visiting so other family members can be more involved in the child's care)[1]
7. What are the best ways to ensure children and families get and understand the information they need, in order to make informed decisions, around the time of diagnosis, during treatment, at the end of treatment and after treatment has finished?
8. What impact does cancer and treatment have on the lives of children and families after treatment, and in the long-term; what are the best ways to help them to overcome these impacts to thrive and not just survive?
9. How can we make more accessible treatments that are closer to home, in shared care hospitals?[1]
10. What is the relationship between chronic fatigue syndrome, fibromyalgia, chronic pain and treatment for childhood cancer? (Fibromyalgia is a long-term condition that causes pain all over the body.)

11. What are the best ways to provide emotional support for children and their families 1) around the time of diagnosis, 2) during treatment and 3) after treatment (including survivors who are now adults)?[1]
12. What are the best ways to reduce, predict and manage the side-effects of treatment for children (including life threatening side-effects)?
13. How can transition (moving) from child into adult services be improved for young people who had cancer as a child?
14. What is the psychological and social impact of cancer and treatment on children and their families during treatment and in the long-term; what factors affect these impacts?
15. How common are the different long-term effects of childhood cancer treatment, how do they change across the lifespan, can we predict them and how can they best be prevented, detected and/or treated?
16. What are the best ways to support the emotional wellbeing of professionals who care for children with cancer and their families?
17. During and after treatment, what issues prevent or encourage physical activity, which interventions are most effective and what should be measured to assess effectiveness?
18. What are the best ways of making sure people who had cancer as a child receive the information they need about the long-term effects of cancer and treatment?
19. What fertility preservation options work best for children and teenagers with cancer?
20. What are the long-term effects of additional medications children with cancer may receive (such as antibiotics, pain killers, laxatives) and how can these effects be reduced?
21. What are children's and survivors' experiences of the side-effects and long-term effects of cancer treatment?
22. How can experiences of having a Hickman line be improved for children with cancer? (A Hickman line is a small tube which is inserted into a vein so that treatments can be given, and blood taken without the repeated need to access veins with a needle. The Hickman line can stay in place for several months.)
23. What are the best ways to support children as they get older, and their needs change, to understand and take responsibility for their health, and to live with the long-term effects of cancer and treatment?

[1] These questions were in the Top 5 research priorities identified by children and young people.

[2] This question was originally not mapped onto the question about emotional support from children and young people, but the workshop participants decided that this question was related as it includes emotional support as well as other types of support.

For peer review only - http://bmjopen.bmj.com/site/about/guidelines.xhtml

DISCUSSION

The Children's Cancer PSP brought together children, survivors, families, and professionals to prioritise research questions on childhood cancer. The Top 10 priorities provide a resource to inform research funding decisions in government and charitable organisations. The top priority is, 'Can we find effective and kinder (less burdensome, more tolerable, with fewer short and long-term effects) treatments for children with cancer, including relapsed cancer?' This question was ranked as top in the shortlisting survey by all three respondent groups (patients/survivors, parents/relatives/friends, and professionals) and placed at number 1 from the start of workshop by all three discussion groups. This reflects shared priorities of continuing to improve cure rates whilst minimising treatment toxicity. The Top 10 priorities reflect the breadth of the cancer experience, including diagnosis, relapse, hospital experience, support during/after treatment and the long-term impact. Priorities highlight the need for research strategies to be holistic in their approach rather than solely driven by biological and drug intervention research. It is now critical that funders and researchers ensure future research focuses on what is important to children, survivors, families and professionals.[9]

A number of cancer-related PSPs exist, including one in Canada also focusing on childhood cancer (https://www.jla.nihr.ac.uk/priority-setting-partnerships/pediatric-cancer/top-10-priorities.htm). The top priority for the Canadian PSP is preventing and managing treatment-related long-term effects which links to the top priority of our PSP and finding 'kinder' treatments. Both Top 10 lists feature similar questions on relapse, prevention/detection and questions about psychosocial impact and support. There is an increasing drive to focus on both physical and psychological health during and after cancer. It is already recognised that a cancer diagnosis has serious implications for children and young people's mental health during and after treatment,[10 11] but this has yet to be systematically investigated, and how best to provide support remains unknown. Psychological support was the top priority in the Teenage and Young Adult Cancer PSP.[12]

Challenges, strengths and limitations

The anticipated timeline for this project was two years, it took three. This delay was partly due to the Covid-19 pandemic. The project was resource intensive, requiring input from all steering group members. The challenge of involving professionals with full schedules, and parent/patient representatives with many concerns and commitments, was amplified by the pandemic, and our progress reflected this.

The scope of the PSP was intentionally broad to reflect the heterogeneity of childhood cancer, and variation in treatment and experience. This generated a significant workload when sorting and summarising diverse questions, and subsequent literature searching to verify uncertainties.

Engaging with children extended the project timescale; this work had to be carefully planned to ensure our methods were accessible and appropriate. Plans for face-to-face work were revised due to pandemic restrictions. Few priority setting exercises have involved many children and young people.[6 13] Previous PSPs have reflected that they were unable to engage with children as they wished, due to lack of time and resources.[14] It was of utmost importance to our steering group that children's voices were heard. We consider this aspect of our PSP a success: time and resources invested in engaging with children were worthwhile. Overall, questions from children reflected similar themes as those from adult participants, but there were some additional elements that featured as higher priority for children, such as having treatments closer to home and improving the hospital experience. In the final workshop, participants wanted children's voices to be heard, resulting in all five of the top priorities identified by children being reflected in the Top 10.

For peer review only - http://bmjopen.bmj.com/site/about/guidelines.xhtml

The use of the rigorous and transparent JLA methodology enhances the validity of the process and results. The response from parents/carers to both surveys was high and parent and patient representatives were involved in shaping the project from the outset, as members of the steering group. Their input was key; for example, they helped to ensure the surveys were presented in a user-friendly format and appropriate routes to dissemination were used. Parent/patient representatives reported a positive experience of being involved in the steering group, *"I wanted to be involved with the PSP because of the exciting opportunity to contribute towards future research topics in childhood cancer, bringing the voice of childhood cancer survivors from a service user perspective and advocating for the cohort. I have found the experience to be extremely positive and engaging. I feel that my presence is valued, and my contributions have been acknowledged and implemented throughout the process."*

Absent voices must be considered as a limitation. Of note, the majority of respondents described themselves as White. The priorities therefore represent the views of the majority, White population, which has been observed in other PSPs.[15] Males were also underrepresented. We did not ask in the surveys whether respondents have a disability (whether resulting from treatment or not) and so cannot understand what impact this might have had on prioritisation.

Primary care has an important role in the care of children with cancer from diagnosis into survivorship[16]. There was a primary care representative on the steering group and at the final workshop, but none responded to the initial survey, and only one to the shortlisting survey. The voices of these professionals are absent from the questions collected.

IMPLICATIONS AND DISSEMINATION

The Top 10 have been circulated on social media and via supporter newsletters/websites by the PSP funding charities and our Partners. Dissemination includes publication of a final report with an associated launch event, peer-reviewed publications, and conference presentations. We will report the detail of our engagement with children in a separate publication and are working with the JLA to develop guidance for future PSPs.

Our aspiration is that these prioritised questions will help to direct and shape future research. The uncertainties identified are the outcome of a systematic and transparent process and provide funders with clear guidance on the highest priorities for future research, voted on by end-users of research. Identifying clear areas for future research allows research funders to target funds effectively and inform fundraising activities. We plan to hold a meeting with funders to promote the priorities and encourage funding calls focused on the priority areas.

When selecting questions to be included in the Top 10, workshop participants intentionally opted for broad questions, to capture the widest range of issues. This is common in JLA PSPs, the questions therefore reflect broad topic areas for research; further refinement is required to transform topics into answerable research questions.[17] This PSP also demonstrates that where sufficient expertise and resources are available, involvement of young children can be achieved. Therefore, funding guidance should encourage applicants to undertake such work.

Some questions submitted were outside the scope of the PSP and were removed. Many suggested a knowledge gap. The steering group considered these questions to be important and is determined to ensure these submissions are not 'lost'. We will look at how these questions, statements and service enquiries can be best used to improve information signposting. Questions were submitted regarding disparity in funding between childhood and 'adult' cancers. These questions were removed, as they are not amenable to research, but we intend to share them through a commentary piece, as they

For peer review only - http://bmjopen.bmj.com/site/about/guidelines.xhtml

reflected strong opinions and perceptions that would benefit from further exploration and articulation.

CONCLUSION

We have identified shared research priorities for children's cancer using a rigorous, person-centred approach involving stakeholders not typically involved in setting the research agenda, including children. Resulting questions reflect the breadth of the cancer experience for children and families, including diagnosis, relapse, hospital experience, support during and after treatment and the long-term impact of cancer. These must inform funding of future research, with priority questions evidenced by researchers.

AUTHOR CONTRIBUTIONS

All authors were part of the Children's Cancer Priority Setting Partnership steering group or coordinating team and made substantive contributions to the conduct of the study, overseeing all aspects of the work. All authors contributed to protocol design, survey refining, data cleaning and refining questions submitted in the initial survey. The project was managed by SA, FG (guarantor), BP, RH and JG. SA, FG, JM, SM, LL, KM, RRB were part of a subgroup overseeing engagement with children throughout the PSP process. Specific contributions included: survey design (SA), coding the survey submissions (FG, SA), searching and checking uncertainties (SA, BP), managing data entry (SA). All authors reviewed and approved the final version of this paper.

ACKNOWLEDGEMENTS

The Children's Cancer PSP would like to thank everyone who took the time to send in their questions and vote on the importance of them. Thank you also to the children, young people, parents, relatives, and professionals who attended the workshops.

We would like to thank Angela Stewart for providing administrative support to the PSP. We would also like to thank the previous members of the steering group: Martin English, Penelope Hart-Spencer, Charmaine Jagger, and Angela Polanco.

FUNDING STATEMENT

This work was supported by Children's Cancer and Leukaemia Group (CCLG) and Little Princess Trust.

Dr. Julia Chisholm is supported by the Giant Pledge through the Royal Marsden Cancer Charity and this independent research is supported by the National Institute for Health Research (NIHR) Biomedical Research Centre at The Royal Marsden NHS Foundation Trust and the Institute of Cancer Research, London. The views expressed are those of the authors and not necessarily those of the NIHR or the Department of Health and Social Care.

Professor Faith Gibson is supported in-part by the Great Ormond Street NIHR Biomedical Research Centre.

COMPETING INTERESTS

None declared.

PATIENT AND PUBLIC INVOLVEMENT

Patients and/or the public were involved in the design, or conduct, or reporting or dissemination plans of this research. See the Methods section for further details.

For peer review only - http://bmjopen.bmj.com/site/about/guidelines.xhtml

For peer review only

DATA SHARING STATEMENT

Further data regarding the source of original submissions to the survey are available from: https://www.jla.nihr.ac.uk/priority-setting-partnerships/childrens-cancer/

ETHICAL APPROVAL

Ethical approvals are not required for JLA priority setting partnerships as per JLA and National Health Services Patient Safety Agency National Research Ethics Service guidance (https://www.invo.org.uk/posttypepublication/public-involvement-in-research-and-research-ethics-committee-review/).

REFERENCES

1. Cancer Research UK. Children's cancers incidence statistics 2018 [Available from: https://www.cancerresearchuk.org/health-professional/cancer-statistics/childrens-cancers/incidence accessed 20th April 2023.
2. Cancer Research UK. Children's cancers survival statistics 2016 [Available from: https://www.cancerresearchuk.org/health-professional/cancer-statistics/childrens-cancers/survival accessed 20th April 2023.
3. Chalmers I, Atkinson P, Fenton M, et al. Tackling treatment uncertainties together: the evolution of the James Lind Initiative, 2003-2013. *Journal of the Royal Society of Medicine* 2013;106(12):482-91. doi: 10.1177/0141076813493063
4. Crowe S, Fenton M, Hall M, et al. Patients', clinicians' and the research communities' priorities for treatment research: there is an important mismatch. *Research involvement and engagement* 2015;1:2. doi: 10.1186/s40900-015-0003-x
5. James Lind Alliance. The James Lind Alliance Guidebook. Version 10 2021 [Available from: https://www.jla.nihr.ac.uk/jla-guidebook/ accessed 13th April 2023.
6. Postma L, Luchtenberg ML, Verhagen AAE, et al. Involving children and young people in paediatric research priority setting: a narrative review. *BMJ Paediatr Open* 2022;6(1):e001610. doi: 10.1136/bmjpo-2022-001610
7. Aldiss S, Hollis R, Phillips B, et al. Research priorities in children's cancer. Final report of the James Lind Alliance Children's Cancer Priority Setting Partnership 2023 [Available from: https://www.jla.nihr.ac.uk/priority-setting-partnerships/childrens-cancer/ accessed 29th June 2023.
8. Aussems K, Schoemaker CG, Verwoerd A, et al. Research agenda setting with children with juvenile idiopathic arthritis: Lessons learned. *Child: care, health and development* 2022;48(1):68-79. doi: 10.1111/cch.12904
9. Gibson F, Aldiss S. What Are the Consequences of Not Responding to Research Priority Setting Exercises? *Cancer Care Research Online* 2023;3(1):e037. doi: 10.1097/cr9.0000000000000037
10. Berkman AM, Robert RS, Roth M, et al. A review of psychological symptoms and post-traumatic growth among adolescent and young adult survivors of childhood cancer. *J Health Psychol* 2022;27(4):990-1005. doi: 10.1177/1359105320971706
11. Sharkey CM, Espeleta HC, Traino KA, et al. Psychological adjustment outcomes among pediatric brain tumor survivors: A meta-analysis. *Pediatric blood & cancer* 2020;67(10):e28644. doi: 10.1002/pbc.28644
12. Aldiss S, Fern LA, Phillips RS, et al. Research priorities for young people with cancer: a UK priority setting partnership with the James Lind Alliance. *BMJ open* 2019;9(8):e028119. doi: 10.1136/bmjopen-2018-028119
13. Odgers HL, Tong A, Lopez-Vargas P, et al. Research priority setting in childhood chronic disease: a systematic review. *Archives of disease in childhood* 2018;103(10):942-51. doi: 10.1136/archdischild-2017-314631

For peer review only - http://bmjopen.bmj.com/site/about/guidelines.xhtml

14. Morris C, Simkiss D, Busk M, et al. Setting research priorities to improve the health of children and young people with neurodisability: a British Academy of Childhood Disability-James Lind Alliance Research Priority Setting Partnership. *BMJ open* 2015;5(1):e006233. doi: 10.1136/bmjopen-2014-006233
15. Drury NE, Herd CP, Biglino G, et al. Research priorities in children and adults with congenital heart disease: a James Lind Alliance Priority Setting Partnership. *Open Heart* 2022;9(2) doi: 10.1136/openhrt-2022-002147
16. Jain J, Qorri B, Szewczuk MR. The crucial role of primary care providers in the long-term follow-up of adult survivors of childhood cancer. *Cancer Manag Res* 2019;11:3411-18. doi: 10.2147/cmar.S197644
17. Staley K, Crowe S, Crocker JC, et al. What happens after James Lind Alliance Priority Setting Partnerships? A qualitative study of contexts, processes and impacts. *Research involvement and engagement* 2020;6:41. doi: 10.1186/s40900-020-00210-9

Figure 1 Pathway of care included in the project scope

Figure 2 Overview of the Children's Cancer Priority Setting Partnership methodology and results

For peer review only - http://bmjopen.bmj.com/site/about/guidelines.xhtml

Figure 1 Pathway of care included in the project scope

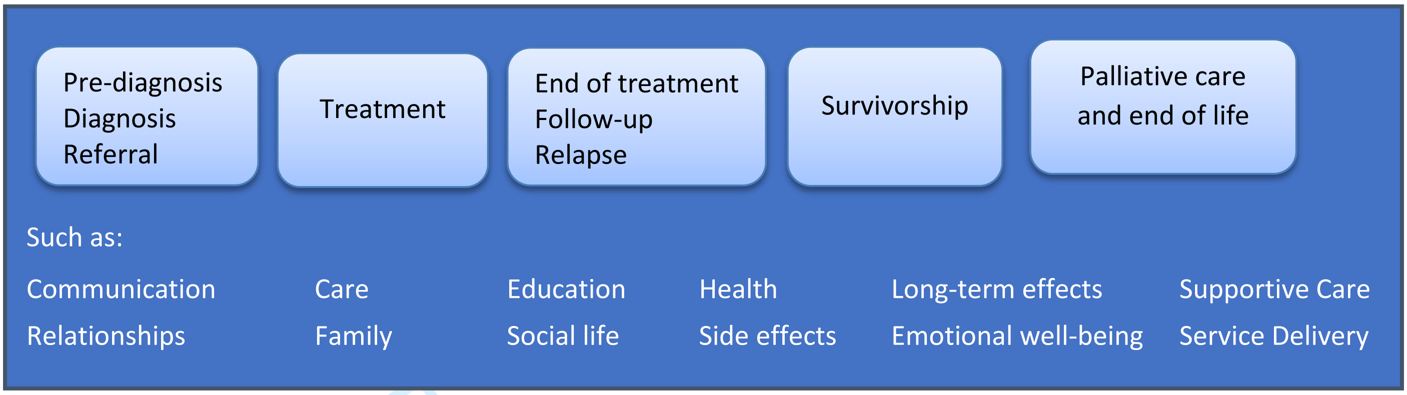

For peer review only

Figure 2 Overview of the Children's Cancer Priority Setting Partnership methodology and results

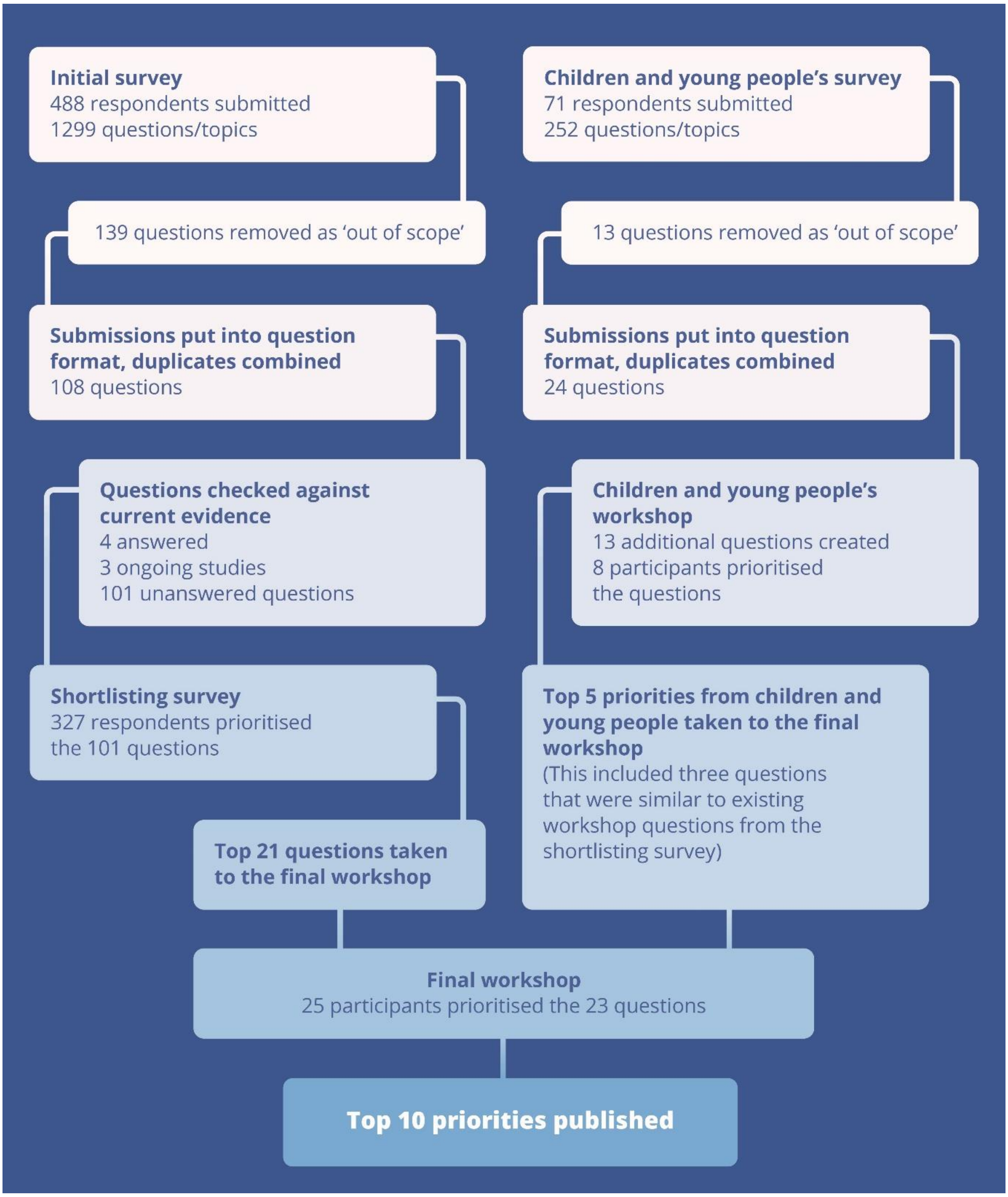

Supplementary material 1 Participant details first survey

	Response	Survivors (number=49)	Parents/relatives/friends/ (number=291)	Professionals (number=148)
**Gender**	Female	41 (84%)	260 (89%)	133 (90%)
	Male	8 (16%)	30 (10%)	14 (9%)
	Use another term	0 (0%)	0 (0%)	0 (0%)
	Prefer not to answer	0 (0%)	1 (0.3%)	1 (1%)
**Trans**	No	48 (98%)	281 (97%)	139 (94%)
	Yes	0 (0%)	0 (0%)	1 (1%)
	Prefer not to answer	0 (0%)	1 (0.3%)	0 (0%)
	Missing data	1 (2%)	9 (3%)	8 (5%)
**Ethnic group**	White	46 (94%)	276 (95%)	135 (91%)
	Asian or Asian British	1 (2%)	4 (1%)	6 (4%)
	Black African, Black Caribbean or Black British	0 (0%)	2 (1%)	2 (1%)
	Mixed/multiple ethnic groups	0 (0%)	4 (1%)	1 (1%)
	Other	2 (4%)	1 (0.3%)	2 (1%)
	Prefer not to answer	0 (0%)	2 (1%)	0 (0%)
	Missing data	0 (0%)	2 (1%)	2 (1%)
**Age (years)**	16-18	6 (12%)	0 (0%)	n/a
	19-24	4 (8%)	1 (0.3%)	3 (2%)
	25-34	18 (37%)	46 (16%)	33 (22%)
	35-44	12 (24%)	127 (44%)	39 (26%)
	45-54	7 (14%)	83 (29%)	46 (31%)
	55-64	1 (2%)	23 (8%)	23 (15%)
	65+	1 (2%)	5 (2%)	1 (1%)
	Prefer not to answer	0 (0%)	1 (0.3%)	1 (1%)
	Missing data	0 (0%)	5 (2%)	2 (1%)
**Country of residence (survivors/parents/relatives/friends) Country of work (professionals)**	England	36 (73%)	241 (83%)	123 (83%)
	Scotland	2 (4%)	12 (4%)	7 (5%)

For peer review only

	Wales	0 (0%)	13 (4%)	6 (4%)
	Northern Ireland	0 (0%)	4 (1%)	3 (2%)
	Other	10 (20%)	20 (7%)	8 (5%)
	Prefer not to answer	1 (2%)	1 (0.3%)	0 (0%)
	Missing data	0 (0%)	0 (0%)	1 (1%)
**Cancer first diagnosed with**	Bone tumour	6 (12%)	7 (2%)	n/a
	Brain or spinal tumour	3 (6%)	35 (12%)	n/a
	Germ cell tumour	0 (0%)	5 (2%)	n/a
	Kidney tumour	2 (4%)	18 (6%)	n/a
	Langerhans Cell Histiocytosis (LCH)	0 (0%)	5 (2%)	n/a
	Leukaemia	20 (41%)	132 (45%)	n/a
	Liver tumour	0 (0%)	1 (0.3%)	n/a
	Lymphoma	8 (16%)	19 (7%)	n/a
	Neuroblastoma	1 (2%)	22 (8%)	n/a
	Retinoblastoma	4 (8%)	14 (5%)	n/a
	Soft tissue sarcoma	4 (8%)	21 (7%)	n/a
	More than one cancer diagnosis	0 (0%)	5 (2%)	n/a
	Not sure	1 (2%)	2 (1%)	n/a
	Other	0 (0%)	5 (2%)	n/a
	Prefer not to answer	0 (0%)	0 (0%)	n/a
**Current situation**	On treatment	1 (2%)	90 (31%)	n/a
	Finished treatment in the last 0 to 12 months	0 (0%)	48 (16%)	n/a
	Finished treatment 1 to 5 years ago	3 (6%)	53 (18%)	n/a
	Finished treatment more than 5 years ago	40 (82%)	29 (10%)	n/a
	On treatment for relapse	2 (4%)	22 (8%)	n/a
	Receiving palliative care	0 (0%)	1 (0.3%)	n/a
	Passed away	n/a	45 (15%)	n/a
	Not sure	1 (2%)	0 (0%)	n/a
	Other	0 (0%)	3 (1%)	n/a

For peer review only

	Prefer not to answer	0 (0%)	0 (0%)	n/a
	Missing data	2 (4%)	0 (0%)	n/a
**Age at diagnosis**	Under 1	2 (4%)	24 (8%)	n/a
	1-3 years	9 (18%)	96 (33%)	n/a
	4-6 years	7 (14%)	66 (23%)	n/a
	7-9 years	9 (18%)	39 (13%)	n/a
	10-12 years	10 (20%)	26 (9%)	n/a
	13-15 years	11 (22%)	26 (9%)	n/a
	Over 16	0 (0%)	5 (2%)	n/a
	Not sure	0 (0%)	2 (1%)	n/a
	Prefer not to answer	0 (0%)	1 (0.3%)	n/a
	Missing data	1 (2%)	6 (2%)	n/a
**Professional group**	Allied health professional	n/a	n/a	49 (33%)
	Nurse	n/a	n/a	45 (30%)
	Doctor	n/a	n/a	27 (18%)
	Education professional	n/a	n/a	17 (11%)
	Social care professional	n/a	n/a	10 (7%)

For peer review only

Supplementary material 2 Participant details children and young people's surveys

	Response	Children and young people with cancer (n=61)	Siblings (n=10)
**Gender**	Male	22 (36%)	5 (50%)
	Female	38 (62%)	5 (50%)
	Prefer not to answer	1 (2%)	0 (0%)
**Country of residence**	England	42 (69%)	6 (60%)
	Scotland	9 (15%)	2 (20%)
	Wales	6 (10%)	2 (20%)
	Northern Ireland	1 (2%)	0 (0%)
	Other	2 (3%)	0 (0%)
	Prefer not to answer	1 (2%)	0 (0%)
**Diagnosis**	Leukaemia	26 (43%)	3 (30%)
	Kidney tumour	7 (11%)	0 (0%)
	Lymphoma	7 (11%)	1 (10%)
	Brain/spinal tumour	5 (8%)	2 (20%)
	Soft tissue sarcoma	4 (7%)	0 (0%)
	Neuroblastoma	3 (5%)	2 (2%)
	Retinoblastoma	2 (3%)	0 (0%)
	Bone tumour	1 (2%)	0 (0%)
	More than one cancer diagnosis	1 (2%)	0 (0%)
	Other	2 (3%)	1 (10%)
	Prefer not to answer	2 (3%)	0 (0%)
	Do not know	1 (2%)	1 (10%)
**Ethnic group* (Children and young people with cancer n=36; Siblings n=7)**	White	31 (86%)	7 (100%)
	Asian or Asian British	1 (3%)	0 (0%)
	Black African, Black Caribbean or Black British	1 (3%)	0 (0%)
	Mixed/multiple ethnic groups	1 (3%)	0 (0%)
	Prefer not to answer	2 (6%)	0 (0%)
**Current situation* (Children and young people with cancer n=36; Siblings n=7)**	On treatment	12 (33%)	3 (43%)
	Finished treatment	23 (64%)	4 (57%)
	Other	1 (3%)	0 (0%)

*not asked in 4-7 year olds survey

For peer review only

Supplementary material 3 Participant details shortlisting survey

	Response	Survivors (number=27)	Parents/relatives/friends/ (number=210)	Professionals (number=90)
**Gender**	Female	23 (85%)	186 (89%)	75 (83%)
	Male	3 (11%)	21 (10%)	14 (16%)
	Use another term	1 (4%)	2 (1%)	0 (0%)
	Prefer not to answer	0 (0%)	0 (0%)	1 (1%)
	Missing data	0 (0%)	1 (0.5%)	0 (0%)
**Trans**	No	26 (96%)	206 (98%)	87 (97%)
	Yes	1 (4%)	0 (0%)	1 (1%)
	Prefer not to answer	0 (0%)	2 (1%)	2 (2%)
	Missing data	0 (0%)	2 (1%)	0 (5%)
**Ethnic group**	White	24 (89%)	199 (95%)	79 (88%)
	Asian or Asian British	1 (4%)	3 (1%)	5 (6%)
	Black African, Black Caribbean or Black British	0 (0%)	0 (0%)	1 (1%)
	Mixed/multiple ethnic groups	1 (4%)	5 (2%)	4 (4%)
	Other	1 (4%)	1 (0.5%)	0 (0%)
	Prefer not to answer	0 (0%)	1 (0.5%)	1 (1%)
	Missing data	0 (0%)	1 (0.5%)	0 (0%)
**Age (years)**	16-18	5 (19%)	1 (0.5%)	0 (0%)
	19-24	9 (33%)	3 (1%)	0 (0%)
	25-34	6 (22%)	24 (11%)	23 (26%)
	35-44	3 (11%)	98 (47%)	26 (29%)
	45-54	3 (11%)	57 (27%)	30 (33%)
	55-64	1 (4%)	19 (9%)	8 (9%)
	65+	0 (0%)	7 (3%)	1 (1%)
	Prefer not to answer	0 (0%)	0 (0%)	2 (2%)
	Missing data	0 (0%)	1 (0.5%)	0 (0%)
**Country of residence (survivors/parents/relatives/friends) Country of work (professionals)**	England	25 (93%)	170 (81%)	78 (87%)

For peer review only

	Scotland	0 (0%)	17 (8%)	6 (7%)
	Wales	0 (0%)	10 (5%)	3 (3%)
	Northern Ireland	0 (0%)	2 (1%)	1 (1%)
	Other	1 (4%)	10 (5%)	1 (1%)
	Prefer not to answer	1 (4%)	1 (0.5%)	1 (1%)
	Missing data	0 (0%)	0 (0%)	0 (0%)
**Cancer first diagnosed with**	Bone tumour	4 (15%)	6 (3%)	n/a
	Brain or spinal tumour	2 (7%)	26 (12%)	n/a
	Germ cell tumour	0 (0%)	3 (1%)	n/a
	Kidney tumour	0 (0%)	10 (5%)	n/a
	Langerhans Cell Histiocytosis (LCH)	0 (0%)	0 (0%)	n/a
	Leukaemia	10 (37%)	113 (54%)	n/a
	Liver tumour	0 (0%)	0 (0%)	n/a
	Lymphoma	4 (15%)	15 (7%)	n/a
	Neuroblastoma	1 (4%)	9 (4%)	n/a
	Retinoblastoma	1 (4%)	8 (4%)	n/a
	Soft tissue sarcoma	3 (11%)	14 (7%)	n/a
	More than one cancer diagnosis	1 (4%)	1 (0.5%)	n/a
	Not sure	0 (0%)	0 (0%)	n/a
	Other	1 (4%)	4 (2%)	n/a
	Prefer not to answer	0 (0%)	0 (0%)	n/a
	Missing data	0 (0%)	1 (0.5%)	n/a
**Current situation**	On treatment	2 (7%)	58 (28%)	n/a
	Finished treatment in the last 0 to 12 months	1 (4%)	21 (10%)	n/a
	Finished treatment 1 to 5 years ago	5 (19%)	60 (29%)	n/a
	Finished treatment more than 5 years ago	19 (70%)	30 (14%)	n/a
	On treatment for relapse	0 (0%)	8 (4%)	n/a
	Receiving palliative care	0 (0%)	2 (1%)	n/a
	Passed away	n/a	26 (12%)	n/a
	Not sure	0 (0%)	0 (0%)	n/a
	Other	0 (0%)	4 (2%)	n/a

	Prefer not to answer	0 (0%)	0 (0%)	n/a
	Missing data	0 (0%)	1 (0.5%)	n/a
**Age at diagnosis**	Under 1	1 (4%)	16 (8%)	n/a
	1-3 years	1 (4%)	63 (30%)	n/a
	4-6 years	6 (22%)	54 (26%)	n/a
	7-9 years	4 (15%)	25 (12%)	n/a
	10-12 years	3 (11%)	26 (12%)	n/a
	13-15 years	10 (37%)	23 (11%)	n/a
	Over 16	2 (7%)	1 (0.5%)	n/a
	Not sure	0 (0%)	0 (0%)	n/a
	Prefer not to answer	0 (0%)	0 (0%)	n/a
	Missing data	0 (0%)	2 (1%)	n/a
**Professional group**	Allied health professional	n/a	n/a	25 (28%)
	Nurse	n/a	n/a	30 (33%)
	Doctor	n/a	n/a	25 (28%)
	Education professional	n/a	n/a	4 (4%)
	Social care professional	n/a	n/a	4 (4%)
	Other	n/a	n/a	2 (2%)

For peer review only

Manuscript title: Research priorities for children's cancer: a James Lind Alliance priority setting partnership

Reporting guideline for priority setting of health research (REPRISE) - checklist

Tong A, Synnot A, Crowe S, Hill S, Matus A, Scholes-Robertson N, Oliver S, Cowan K, Nasser M, Bhaumik S, Gutman T, Baumgart A, Craig JC. Reporting guideline for priority setting of health research (REPRISE). BMC Med Res Methodol. 2019 Dec 28;19(1):243. doi: 10.1186/s12874-019-0889-3.

	**REPRISE reporting item**	**REPRISE descriptor and/or examples**	**Reported on Page #/in section**
**A**	**Context and scope**		
1	Define geographical scope	Global, regional, national, city, local area, institutional/organizational level, health service	Title, abstract and Introduction
2	Define health area, field, focus	Disease or condition specific, interventions, healthcare delivery, health system	Methods: Scope
3	Define the intended beneficiaries	This may include the general population or a specific population based on demographic (age, gender), clinical (disease, condition), or other characteristics who may benefit from the research	Introduction/discussion
4	Define the target audience of the priorities	Policy makers, funders, researchers, industry or others who have the potential to implement the priorities identified	Introduction/discussion
5	Identify the research area	Public health, health services research, clinical research, basic science	Methods: Scope
6	Identify the type of research questions	Etiology, diagnosis, prevention, treatment (interventions), prognosis, health services, psychosocial, behavioral and social science, economic evaluation, implementation; this may not be pre-defined	Methods: Scope and process
7	Define the time frame	Interim, short-term, long-term priorities, plans to revise and update	Not reported as no current plans to update, this will partly depend on whether questions are answered in future.
**B**	**Governance and team**		

8	Describe the selection and structure of the leadership and management team	Those responsible for initiating, developing, and guiding the process for priority setting, and examples of structures include; Steering Committee, Advisory Group, Technical Experts	Methods: Set-up
9	Describe the characteristics of the team	Stakeholder group or role, institutional affiliations, country or region, demographics (e.g. age sex), discipline, experience, expertise	Methods: Set-up
10	Describe any training or experience relevant to conducting priority setting	Consultants or advisers, members with experience or skills relevant to the conducting priority-setting e.g. qualitative methods, surveys, facilitation	Methods: Set-up
**C**	**Framework for priority setting**		
11	State the framework used (if any)	James Lind Alliance, COHRED, CHNRI, Dialogue Model, no framework (general research priority setting)	Methods
**D**	**Stakeholders or participants**		
12	Define the inclusion criteria for stakeholders involved in priority-setting	Patients, caregivers, general community, health professionals, researchers, policy makers, non-governmental organizations, government, industry; specific groups including vulnerable and marginalized populations	Methods: Process
13	State the strategy or method for identifying and engaging stakeholders	Partnership with organizations, social media, recruitment through hospitals	Methods: Process
14	Indicate the number of participants and/or organizations involved	Number of individuals and organizations, include number by stakeholder group	Results
15	Describe the characteristics of stakeholders	Stakeholder group, demographic characteristics, areas of interest and expertise, discipline, affiliations	Results and supplementary material 1,2 and 3
16	State if reimbursement for participation was provided	Cash, vouchers, certificates, acknowledgement; what purpose e.g. travel, accommodation, honorarium	PPI section
**E**	**Identification and collection of research priorities**		
17	Describe methods for collecting initial priorities	Methods e.g. Delphi survey, surveys, nominal group technique, interviews, focus groups, meetings, workshops; prioritization e.g. voting, ranking; mode e.g. face-to-face, online;	Methods: Process

		may be informed by evidence e.g. systematic reviews, reviews of guidelines/other documents, health technology assessment	
18	Describe methods for collating and categorizing priorities	Taxonomy or other framework used to organize, summarise, and aggregate topics or questions	Methods: Process
19	Describe methods and reasons for modifying (removing, adding, reframing) priorities	Based on scope, clarity, definition, duplication, other criteria	Methods: Process
20	Describe methods for refining or translating priorities into research topics or questions	Reviewed by Steering Committee or project team	Methods: Process
21	Describe methods for checking whether research questions or topics have been answered	Systematic reviews, evidence mapping, consultation with experts	Methods: Process
22	Describe number of research questions or topics	Number of priorities at each stage of the process	Methods: Results
**F**	**Prioritization of research topics/questions**		
23	Describe methods and criteria for prioritizing research topics or questions	Methods e.g. Delphi survey, surveys, nominal group technique, interviews, focus groups, meetings, workshops;<br>Prioritization e.g. voting, ranking;<br>Mode e.g. face-to-face, online;<br>Criteria e.g. need, feasibility, novelty, equity	Methods: Process
24	State the method or threshold for excluding research topics/questions	Thresholds for ranking scores, proportions, votes; other criteria	Methods: Process
**G**	**Output**		

25	State the approach to formulating the research priorities	Area, topic, questions, PICO (population, intervention, comparator, outcome)	Methods: Process
**H**	**Evaluation and feedback**		
26	Describe how the process of prioritization was evaluated	Survey, workshop	Results of JLA feedback surveys from the steering group and workshop are not reported due to word limits.<br>Feedback from a patient representative on steering group is included in the discussion section.
27	Describe how priorities were fed back to stakeholders and/or to the public; and how feedback (if received) was addressed and integrated	Public meetings or workshop, newsletters, website, email, online presentations	Implications and dissemination.
**I**	**Implementation**		
28	Outline the strategy or action plans for implementing priorities	Communication with target audience, via policies and funding	IMPLICATIONS AND DISSEMINATION
29	Describe plans, strategies, or suggestions to evaluate impact	Integration in decision-making, funding allocation, review of relevant documents<br><br>Again, this may be something that Steering Groups build into their earlier discussions about dissemination and implementation.	The James Lind Alliance try to track impact following PSPs (e.g. funding calls relating to PSP priorities). We have not reported this due to word limits.

J	**Funding and conflict of interest**		
30	State sources of funding	Name sources of funding for the priority-setting exercise; if relevant include the budget and/or cost	Funding statement
31	Declare any conflicts or competing interests	State any conflicts of interest that may be at an individual level and/or at a contextual level (e.g. political issues, controversies) that may affect the process, output or implementation.	Competing interests statement

For peer review only

BMJ Open

Research priorities for children's cancer: a James Lind Alliance priority setting partnership in the United Kingdom

Journal:	*BMJ Open*
Manuscript ID	bmjopen-2023-077387.R1
Article Type:	Original research
Date Submitted by the Author:	08-Nov-2023

Complete List of Authors:	Aldiss, Susie; University of Surrey, School of Health Sciences<br>Hollis, Rachel; Leeds Children's Hospital<br>Phillips, Bob; Leeds Teaching Hospitals NHS Trust, Department of Paediatric Haematology and Oncology; University of York, Hull-York Medical School and Centre for Reviews and Dissemination<br>Ball-Gamble, Ashley; Children's Cancer and Leukaemia Group,<br>Brownsdon, Alex; Patient Representative on the Children's Cancer Priority Setting Partnership steering group<br>Chisholm, Julia; The Royal Marsden NHS Foundation Trust; Institute of Cancer Research Sutton<br>Crowther, Scott; Parent Representative on the Children's Cancer Priority Setting Partnership steering group<br>Dommett, Rachel; Bristol Royal Hospital for Children<br>Gower, Jonathan; James Lind Alliance, National Institute for Health Research Evaluation, Trials and Studies Coordinating Centre<br>Hall, Nigel; Southampton Children's Hospital; University of Southampton Faculty of Medicine, University Surgery Unit<br>Hartley, Helen; Alder Hey Children's NHS Foundation Trust<br>Hatton, Jenni; University Hospitals Birmingham NHS Foundation Trust<br>Henry, Louise; The Royal Marsden NHS Foundation Trust<br>Langton, Loveday; Parent Representative on the Children's Cancer Priority Setting Partnership steering group<br>Maddock, Kirsty; Leeds Children's Hospital<br>Malik, Sonia; Young Lives Vs Cancer<br>McEvoy, Keeley; Leeds Children's Hospital, Medical Needs Teaching Service<br>Morgan, Jessica; Leeds Teaching Hospitals NHS Trust, Department of Paediatric Haematology and Oncology; University of York, Centre for Reviews and Dissemination<br>Morris, Helen; University Hospitals Bristol NHS Foundation Trust<br>Parke, Simon; Royal Devon and Exeter NHS Foundation Trust<br>Picton, Sue; Leeds Children's Hospital<br>Reed-Berendt, Rosa; Great Ormond Street Hospital for Children NHS Foundation Trust, Psychological Services<br>Saunders, Dan; The Christie NHS Foundation Trust<br>Stewart, Andy; Parent Representative on the Children's Cancer Priority Setting Partnership Steering Group<br>Tarplee-Morris, Wendy; The Little Princess Trust<br>Walsh, Amy; Parent Representative on the Children's Cancer Priority Setting Partnership Steering Group<br>Watkins, Anna; Parent Representative on the Children's Cancer Priority

	Setting Partnership steering group<br>Weller, David; The University of Edinburgh Edinburgh Medical School<br>Gibson, Faith; University of Surrey, School of Health Sciences; Great Ormond Street Hospital For Children NHS Foundation Trust, ORCHID Centre for Outcomes and Experience Research in Child Health, Illness and Disability
**Primary Subject Heading**:	Oncology
Secondary Subject Heading:	Paediatrics
Keywords:	Paediatric oncology < PAEDIATRICS, Patient Participation, Surveys and Questionnaires, Paediatric oncology < ONCOLOGY

SCHOLARONE™
Manuscripts

I, the Submitting Author has the right to grant and does grant on behalf of all authors of the Work (as defined in the below author licence), an exclusive licence and/or a non-exclusive licence for contributions from authors who are: i) UK Crown employees; ii) where BMJ has agreed a CC-BY licence shall apply, and/or iii) in accordance with the terms applicable for US Federal Government officers or employees acting as part of their official duties; on a worldwide, perpetual, irrevocable, royalty-free basis to BMJ Publishing Group Ltd ("BMJ") its licensees and where the relevant Journal is co-owned by BMJ to the co-owners of the Journal, to publish the Work in this journal and any other BMJ products and to exploit all rights, as set out in our licence.

The Submitting Author accepts and understands that any supply made under these terms is made by BMJ to the Submitting Author unless you are acting as an employee on behalf of your employer or a postgraduate student of an affiliated institution which is paying any applicable article publishing charge ("APC") for Open Access articles. Where the Submitting Author wishes to make the Work available on an Open Access basis (and intends to pay the relevant APC), the terms of reuse of such Open Access shall be governed by a Creative Commons licence – details of these licences and which Creative Commons licence will apply to this Work are set out in our licence referred to above.

Other than as permitted in any relevant BMJ Author's Self Archiving Policies, I confirm this Work has not been accepted for publication elsewhere, is not being considered for publication elsewhere and does not duplicate material already published. I confirm all authors consent to publication of this Work and authorise the granting of this licence.

For peer review only

Research priorities for children's cancer: a James Lind Alliance priority setting partnership in the United Kingdom

Susie Aldiss, Rachel Hollis, Bob Phillips, Ashley Ball-Gamble, Alex Brownsdon, Julia Chisholm, Scott Crowther, Rachel Dommett, Jonathan Gower, Nigel Hall, Helen Hartley, Jenni Hatton, Louise Henry, Loveday Langton, Kirsty Maddock, Sonia Malik, Keeley McEvoy, Jess Morgan, Helen Morris, Simon Parke, Sue Picton, Rosa Reed-Berendt, Dan Saunders, Andy Stewart, Wendy Tarplee-Morris, Amy Walsh, Anna Watkins, David Weller, Faith Gibson

Susie Aldiss, Research Fellow, School of Health Sciences, University of Surrey, Guildford, UK.

Rachel Hollis, PSP Lead, Honorary Nurse Advisor, Leeds Children's Hospital, Leeds, UK

Bob Phillips, Professor and Consultant in Paediatric Oncology, Department of Paediatric Haematology and Oncology, Leeds Teaching Hospitals NHS Trust, Leeds and Hull-York Medical School and Centre for Reviews and Dissemination, University of York, York, UK

Ashley Ball-Gamble, Chief Executive Officer, Children's Cancer and Leukaemia Group, Leicester, UK

Alex Brownsdon, Patient Representative on the Children's Cancer Priority Setting Partnership steering group, London, UK

Julia Chisholm, Consultant in Paediatric and Adolescent Oncologist, The Royal Marsden NHS Foundation Trust and Institute of Cancer Research, Sutton, UK

Scott Crowther, Parent Representative on the Children's Cancer Priority Setting Partnership steering group, Coventry, UK

Rachel Dommett, Consultant in Paediatric Leukaemia and Aftercare, Bristol Royal Hospital for Children, Bristol, UK

Jonathan Gower, James Lind Alliance Advisor, The James Lind Alliance, National Institute for Health Research Evaluation, Trials and Studies Coordinating Centre, University of Southampton, Southampton, UK

Nigel Hall, Consultant Paediatric Surgeon, Southampton Children's Hospital; Associate Professor of Paediatric Surgery, University of Southampton, Southampton, UK

Helen Hartley, Specialist Paediatric Physiotherapist - Acute Neurosciences/ Oncology, Alder Hey Children's NHS Foundation Trust, Liverpool, UK

Jenni Hatton, Advanced Pharmacist Clinical Oncology, University Hospitals Birmingham NHS Foundation Trust, Birmingham, UK

Louise Henry, Senior Specialist Dietitian (Paediatrics and TYA), The Royal Marsden NHS Foundation Trust, Sutton, UK

Loveday Langton, Parent Representative on the Children's Cancer Priority Setting Partnership steering group, London, UK

Kirsty Maddock, Speech & Language Therapist, Leeds Children's Hospital, Leeds, UK

Sonia Malik, Head of Policy and Influencing, Young Lives vs Cancer, London, UK

For peer review only - http://bmjopen.bmj.com/site/about/guidelines.xhtml

Keeley McEvoy, Assistant Headteacher, Medical Needs Teaching Service, Leeds Children's Hospital, Leeds, UK

Jess Morgan, Senior Research Fellow in Paediatric Oncology, Department of Paediatric Haematology and Oncology, Leeds Teaching Hospitals NHS Trust, Leeds and Centre for Reviews and Dissemination, University of York, York, UK

Helen Morris, Children's Cancer Network Lead Nurse, Children, Teenage and Young Adult Cancer Operational Delivery Network, South West, Bristol, UK

Simon Parke, Consultant Paediatrician, Royal Devon and Exeter NHS Foundation Trust, UK

Sue Picton, Consultant Paediatric and Adolescent Oncologist, Leeds Children's' Hospital/ Martin House Hospice, UK

Rosa Reed-Berendt, Clinical Psychologist, Psychological Services, Great Ormond Street Hospital for Children NHS Foundation Trust, London, UK.

Dan Saunders, Consultant in Clinical Oncology, The Christie NHS Foundation Trust, Manchester, UK

Andy Stewart, Parent Representative on the Children's Cancer Priority Setting Partnership Steering Group, Perth, UK

Wendy Tarplee-Morris, Co-founder and Director of Service and Impact, The Little Princess Trust, Hereford, UK

Amy Walsh, Parent Representative on the Children's Cancer Priority Setting Partnership Steering Group, Keswick, UK

Anna Watkins, Parent Representative on the Children's Cancer Priority Setting Partnership Steering Group, London, UK

David Weller, Professor of General Practice, University of Edinburgh, Edinburgh, UK

Faith Gibson, ORCHID Centre for Outcomes and Experience Research in Child Health, Illness and Disability, Great Ormond Street Hospital for Children NHS Foundation Trust, London, UK and School of Health Sciences, University of Surrey, Guildford, UK.

Corresponding author:

Susie Aldiss, School of Health Sciences, Kate Granger Building, 30 Priestley Road, Surrey Research Park, Guildford, GU2 7YH, UK. s.aldiss@surrey.ac.uk

Keywords: Cancer, children, research priorities, James Lind Alliance, patient and public involvement

For peer review only - http://bmjopen.bmj.com/site/about/guidelines.xhtml

ABSTRACT

Objectives

To engage children who have experienced cancer, childhood cancer survivors, their families, and professionals to systematically identify and prioritise research questions about childhood cancer to inform the future research agenda.

Design

James Lind Alliance Priority Setting Partnership.

Setting

UK health service and community.

Methods

A steering group oversaw the initiative. Potential research questions were collected in an online survey, then checked to ensure they were unanswered. Shortlisting via a second online survey identified the highest priority questions. A parallel process with children was undertaken. A final consensus workshop was held to determine the Top 10 priorities.

Participants

Children and survivors of childhood cancer, diagnosed before age 16, their families, friends, and professionals who work with this population.

Results

Four hundred and eighty-eight people submitted 1299 potential questions. These were refined into 108 unique questions; four were already answered and three were under active study, therefore removed. Three hundred and twenty-seven respondents completed the shortlisting survey. Seventy-one children submitted questions in the children's surveys, eight children attended a workshop to prioritise these questions. The Top 5 questions from children were taken to the final workshop where 23 questions in total were discussed by 25 participants (young adults, carers and professionals). The top priority was, 'Can we find effective and kinder (less burdensome, more tolerable, with fewer short- and long-term effects) treatments for children with cancer, including relapsed cancer?'

Conclusions

We have identified research priorities for children's cancer from the perspectives of children, survivors, their families, and the professionals who care for them. Questions reflect the breadth of the cancer experience, including diagnosis, relapse, hospital experience, support during/after treatment and the long-term impact of cancer. These should inform funding of future research as they are the questions that matter most to the people who could benefit from research.

For peer review only - http://bmjopen.bmj.com/site/about/guidelines.xhtml

ARTICLE SUMMARY

Strengths and limitations of this study

- We made use of the well-established and transparent James Lind Alliance methodology and clearly describe the process and decision making which led to the final Top 10 research priorities;
- The process followed ensures that these priorities came directly from those who are the most affected by childhood cancer but rarely influence the research agenda;
- We ensured the priorities of patients/survivors, parents/relatives/friends and professionals were given equal weighting at the interim priority setting stage;
- We used innovative methods to hear directly from children about their priorities for future research through surveys and a workshop specifically designed for them;
- Underrepresented groups in the survey submissions included people from minority ethnic groups, males and primary health care professionals.

Word count = 4425

For peer review only - http://bmjopen.bmj.com/site/about/guidelines.xhtml

For peer review only

INTRODUCTION

Annually there are around 1,800 new cases of cancer in children in the UK (1). While research over the last four decades has dramatically increased the overall five-year survival rate for all childhood cancers to around 84% (2) further research is needed to not only improve outcomes for all types of cancer, but to support all children to live long, healthy and happy lives.

Historically, topics of healthcare research in children's cancer have been driven by perspectives of researchers and the pharmaceutical industry, meaning what is most important to children, survivors, their families and the professionals who care for them, has sometimes been overlooked. Prioritising areas for research as identified by children and carers is crucial. There is increasing evidence that research questions and outcomes prioritised by professionals may not be aligned to those experiencing the disease (3). Patients and carers tend to prioritise non-drug treatment research while ongoing research strategies are dominated by drug evaluations (4). This mismatch in priorities is particularly relevant for children due to their unique physiological and psychosocial status and relative rarity of cancer. Increasingly, research funders are asking if proposed research is a priority for patients.

The James Lind Alliance (JLA) is a non-profit making initiative bringing together patients, carers and professionals in Priority Setting Partnerships (PSPs) focusing on specific health conditions http://www.jla.nihr.ac.uk/priority-setting-partnerships/). JLA PSPs identify and prioritise unanswered questions, so researchers and research funders are aware of the issues that matter most to those who could benefit from that research (5).

In 2019, Children's Cancer and Leukaemia Group (CCLG; https://www.cclg.org.uk/) and The Little Princess Trust (https://www.littleprincesses.org.uk/) partnered with the JLA on the Children's Cancer PSP. One of our primary goals was to prioritise the voice of children about what research should be undertaken. Previous PSPs have sought to involve children and young people, but in the final reporting it is evident that few children, especially young children, had been engaged through the process (6). We recognised the challenges of engaging with these populations, in terms of reach and accessibility of information, and determined we would invest time and resources, in exploring and resolving any challenges that could impact on participation.

Following the JLA methodology, we aimed to conduct a UK-wide research prioritisation exercise for childhood cancer to inform decisions of research funders and support the case for research in this underserved group (7).

METHODS

Methodology followed the JLA process (5) the protocol is available from: https://www.jla.nihr.ac.uk/priority-setting-partnerships/childrens-cancer/.

Set-up

Project management

There was a coordinating team of four researchers, nurses and clinicians. An expert steering group (all co-authors) oversaw the project, approved aims/objectives, survey materials, contributed to data analysis and summary question formation, and provided expert opinions for evidence checking. The steering group included parents of a child with cancer (n=5); an adult survivor of childhood cancer; a range of professionals reflecting the multidisciplinary nature of the care of children with cancer including: a teacher, General Practitioner (GP), surgeon, pharmacist, dietitian, speech and

For peer review only - http://bmjopen.bmj.com/site/about/guidelines.xhtml

language therapist, clinical psychologist, physiotherapist, nurses (n=2), doctors (n=6) and representatives from the third sector (n=3), including the charities funding the project. The JLA chair (JG) provided neutral facilitation of meetings. The steering group identified potential partners, mainly children's cancer charities and professional networks, who were approached to assist with survey dissemination.

Scope

This project focused on cancer and cancer-like conditions in children aged 0 to <16 at initial diagnosis. The scope, kept intentionally broad, included questions on any aspect of the cancer experience (Figure 1).

Our aim was, *'To identify gaps and unanswered questions in research about children's cancer from patients, carers and professionals' perspectives and then prioritise those that these groups agree are the most important for research to address.'*

Process

Figure 2 summarises the complete process.

Stage 1a – Gathering questions – initial survey

The survey was developed by the steering group and built using Qualtrics software. It was piloted with eight adult survivors of childhood cancer, nine parents and two professionals outside the steering group and adapted to incorporate their feedback. The survey launched on 9/9/2020 and closed on 8/1/2021. The following groups were invited to participate:

- People diagnosed with cancer before their 16th birthday;
- Relatives/friends/partners/carers of someone diagnosed with cancer before their 16th birthday;
- Professionals involved in diagnosing or treating children who have cancer or had cancer under 16;
- Professionals involved in the care of children who have cancer or had cancer under 16 and/or their families.

Respondents could submit up to eight questions about any aspect of children's cancer they considered important and unanswered. Basic demographic data were requested, and a box was available for free-text comments. Partners promoted the survey through websites, social media, newsletters, and email.

Stage 1b – Gathering questions from children and young people

A subgroup of the steering group was established to focus on our engagement with children. This consisted of two researchers, a teacher, doctor, health play specialist, parent, clinical psychologist, and charity representative. Our initial intention had been to run a series of face-to-face workshops with children to collect questions, this was not possible due to the COVID pandemic until the final workshop in the PSP process.

We determined that the best way to reach children would be through their parents/carers. Three survey versions were built using Qualtrics software, aimed at children of different ages (4-7, 8-12, 13-15 years). Surveys were piloted with three children and young people. They varied in complexity of language used in the introduction and questions, and surveys for young people contained more questions seeking demographic information: participants could complete whichever survey version

For peer review only - http://bmjopen.bmj.com/site/about/guidelines.xhtml

they preferred. Animations were developed to assist parents explain the project and survey to their child(ren) (surveys/animations available here: https://www.jla.nihr.ac.uk/priority-setting-partnerships/childrens-cancer/).

Surveys were launched on 6/9/2021 and closed on 16/11/2021 inviting participants who:

- were diagnosed with cancer before their 16th birthday;
- have a brother or sister with cancer now or who had cancer when they were younger (diagnosed before their 16th birthday);
- have a friend with cancer now or who had cancer when they were younger (diagnosed before their 16th birthday).

Respondents were invited to submit up to eight questions/topics about any aspect of children's cancer they considered important. Surveys were promoted through the PSP's Partners, social media, and posters were sent to all UK Principal Treatment Centres.

Stage 2a - Refining questions from the initial survey

Submitted questions were examined in detail and free-text sections studied for further questions.

Organising the questions

Initial coding was carried out by coordinating team members (SA, FG). Questions were grouped into themes. During coding, potential 'out-of-scope' questions were identified (see Box 1 for criteria used). Identification of out-of-scope questions was an iterative process, checked and agreed by the steering group.

Similar questions were grouped to form summary questions. The aim was to retain the sense of what respondents meant, but in the form of a clear question. Steering group members met online in small groups to review summary questions within their area of expertise/experience, to confirm the grouping of questions, and wording of each summary question. The steering group reviewed the whole summary question list.

Box 1 Out-of-scope question categories and examples

1. The question was ambiguous, was interpreted in different ways by steering group members and the meaning could not be resolved following discussion:
   'Remaining scar tissue'
   'How research is going'

2. Questions not answerable by research:
   'Why does paediatric cancer research receive so very little funding?'
   'Who is present when you give the diagnosis'.

3. Questions submitted by people whose experience was not of childhood cancer as defined by our project scope - there were a few parent respondents whose child was over 16 at diagnosis. These questions were checked to verify that all the themes within them had been covered by 'in scope' questions.

Evidence searching

For peer review only - http://bmjopen.bmj.com/site/about/guidelines.xhtml

Searches were undertaken to identify questions answered by existing evidence. A search strategy was agreed with the steering group (see question verification form: https://www.jla.nihr.ac.uk/priority-setting-partnerships/childrens-cancer/). Searches were carried out by SA in January-May 2022. Searches were limited to evidence published in the last five years (since January 2017) and focused on evidence gathered from multiple studies (e.g. systematic reviews, qualitative meta-synthesis). Searches were undertaken for ongoing studies which included personal communication with experts in the field and steering group members' knowledge of current research.

Stage 2b – Refining questions from children and young people

The same process was followed for refining questions from the children and young people's surveys. Questions were grouped into themes by SA with support from FG, similar questions were merged, and summary questions created. Out-of-scope questions were removed, if they were unrelated to cancer or were unclear (e.g. 'cost to hospital', 'wildlife'). The subgroup met online to review summary questions and out-of-scope questions, with further checking undertaken via email until agreement was reached.

Stage 3 – Question prioritisation

Shortlisting survey preparation

The steering group discussed whether to take all unanswered questions to the shortlisting survey or shorten the list to make the survey quicker to complete. The group chose not to remove any questions.

To ensure questions were easy to understand, they were reviewed by patient and parent members of the steering group and a health information specialist from one of the funding charities. Questions were simplified following this review and definitions of words added.

Shortlisting survey

The shortlisting survey was created using Qualtrics software, launched 3/8/2022 and closed 30/9/2022. Invitations mirrored the initial survey, and it was publicised using the same methods. Initial survey participants who left contact details were emailed directly.

To shorten the question list, respondents were invited to read the 101 questions and select those that were most important to them. Questions selected were added to their own personal 'shortlist' ready for them to make their final selection of up to 15 questions. Survey fatigue was minimised by randomisation of section order and questions. This randomisation aimed to limit question selection bias, for example always selecting the first or last presented questions.

Questions were grouped into:

1. Side-effects and management
2. Treatment
3. Education
4. Physical activity, play and therapies
5. Long-term effects and follow-up care
6. Communication and information sharing
7. Psychological and social wellbeing
8. Food and nutrition
9. Healthcare delivery

For peer review only

10. Causes of cancer, diagnosis and research

Results were analysed in three groups: 1) patients/survivors, 2) parents/friends/relatives, 3) professionals. This gave equal weight to each group's choices as more parents/friends/relatives took part. Questions were given a rank depending on number of votes and ordered from highest to lowest for each group. The steering group reviewed and compared respondent groups and decided to take the Top 10 questions for each of the three groups to the workshop. This ensured that what was important to each group would be considered and resulted in 21 questions being shortlisted, as some questions were shared priorities.

Stage 4a – Workshop with children and young people

The children and young people's workshop took place in October 2022. The workshop was facilitated by SA and FG following the methodology used by the Juvenile Idiopathic Arthritis PSP (8). Children were given a choice of seven envelopes, each containing questions on a different topic with a total of 31 questions. Topics were:

1. Family, friends, and pets
2. Treatments and medicines
3. Being poorly, side-effects and long-term effects
4. Being in hospital
5. Emotions, worries and getting help or support
6. School and education
7. Getting the information you need.

Each participant chose the topic which was most important to them. Envelopes were opened, and participants placed the questions on the table in groups of most, medium or least important. Participants were invited to add more questions if anything of importance to them was missing. They were given three stickers to vote for their Top 3 questions. Questions were placed in order of most to least votes and a discussion followed to agree the 'Top 5'; these were taken to the final workshop.

Stage 4b – Top 10 Prioritisation

The final prioritisation workshop took place in November 2022. Participants who left their contact details in the survey were invited to attend as were patient and parent representatives on the steering group. Steering group contacts were used to ensure participation from a broad range of professionals across the field.

Prior to the workshop, participants were asked to individually rank the questions in order of importance. The workshop was chaired by JG and supported by two JLA facilitators. Participants were split into three pre-allocated groups ensuring a balance of multi-disciplinary professionals, young adults, and parents/relatives. In each group, participants shared their three highest and lowest ranking questions. Participants were told which questions were in the children's Top 5.

During facilitated discussion, the groups ordered the questions from highest to lowest priority. The ranking from the three groups were combined. In a second session, groups were re-allocated and the combined ranking was discussed. Following this discussion, the group rankings were again collated, and all participants formed one group to debate and agree the Top 10.

Patient and Public Involvement

For peer review only - http://bmjopen.bmj.com/site/about/guidelines.xhtml

Parent and patient representatives were involved as equal members of the steering group and in all stages of the prioritisation process. Patients and carers were survey respondents. Children were included in a parallel process. Young adults and parents/relatives attended the final prioritisation workshop alongside professionals as equal stakeholders. Participants were reimbursed for travel/overnight accommodation costs.

RESULTS

Figure 2 provides an overview of the number of respondents at each stage.

Initial survey

Four-hundred and eighty-eight people submitted 1299 questions. Respondents included 49 (10%) patients/survivors, 291 (60%) parents/relatives/friends and 148 (30%) professionals. Most parents/relative/friends were parents (n=271; 93%), 15 (5%) were relatives and five (2%) friends. Supplementary material 1 shows respondent demographics.

One-hundred and thirty-nine out-of-scope questions were removed; Box 1 illustrates examples. Following the combining of similar questions and rewording to form summary questions, 108 questions remained.

Analysis of uncertainties

Four questions were already answered, and three the focus of ongoing studies. For some questions, no reviews or ongoing studies were identified. If reviews only partly answered a question, these were recorded as unanswered. The steering group discussed all questions ensuring consensus agreement of answered/unanswered questions; 101 questions were unanswered.

Children and young people's surveys

Seventy-one respondents submitted 252 questions/topics. Sixty-one respondents were children and young people who had experienced cancer (aged 3-21) and ten were siblings (aged 4-19). No friends participated. See supplementary material 2 for demographics. For brevity, we refer to submissions as 'questions'; nearly all submissions were not written as questions. Thirteen questions were identified as out-of-scope and removed. Responses were summarised into 24 questions.

Shortlisting survey

Ratings were submitted by 327 respondents. Like the initial survey, the largest respondent group was parents/relatives/friends (64%, n=210; including 197 parents, 10 relatives, three friends), followed by professionals (28%, n=90) and patients/survivors (8%, n=27). See supplementary material 3 for demographics.

Children and young people's workshop

Eight children and young people aged 8-16 attended; three were siblings. Their diagnoses included lymphoma and leukaemia.

During discussion, seven additional questions were created about family, friends, and pets and six were added on topics that were important to participants. The Top 5 are shown in Table 1. Three of the questions were closely aligned to those already going to the final workshop from the shortlisting survey (priorities 2, 4 and 5). For priority 4, the children and young people's version of the question had an extra part about starting treatment in the right place, this version was taken to the final

For peer review only - http://bmjopen.bmj.com/site/about/guidelines.xhtml

workshop. Priorities 1 and 3 from children and young people were new and were added into the list, making 23 questions in total for the final workshop.

Table 1 Children and young people's Top 5 and questions for the final workshop

Rank	Top 5 questions from the children and young people's workshop	Question going to the final workshop from the shortlisting survey
1	How can we make being in hospital a better experience for children and young people? (like having better food, internet, toys, and open visiting so other family members can be more involved in the child's care)	
2	How can we prevent cancer in children and young people?	Why do children develop cancer (including the role that genetics plays) and could it be prevented?
3	How can we make more accessible treatments that are closer to home, in shared care hospitals?	
4	How can we speed up the process of getting diagnosed and starting treatment in the right place?	How can time to diagnosis be improved for children with suspected cancer?
5	What are the best ways to help children and young people with their worries and make them feel happier?	What are the best ways to provide emotional support for children and their families 1) around the time of diagnosis, 2) during treatment and 3) after treatment (including survivors who are now adults)?

Final workshop

Twenty-five participants attended: four young adults who had experienced childhood cancer, five parents and one grandparent of a child who had cancer, and 15 professionals who work with this population. Professional roles varied and included nurses, doctors, a social worker, health play specialist, dietitian, clinical psychologist, physiotherapist and chaplain. One participant was a steering group member.

Top 10 Prioritisation strategies

Although the three groups worked independently, they all applied similar prioritisation strategies:

Ensuring children's views were represented

All groups wanted to ensure the Top 10 questions included most, if not all, questions from the children's Top 5. When the groups were told which questions were important to children, those question cards were picked out and moved up the ranking. Most of these questions remained in the Top 10, or just outside, for the duration of the discussions.

For peer review only - http://bmjopen.bmj.com/site/about/guidelines.xhtml

Opting for questions that could include other questions/overlap

Groups considered which questions overlapped and could cover other questions. For example, 'Can we find effective and kinder (less burdensome, more tolerable, with fewer short and long-term effects) treatments for children with cancer, including relapsed cancer?' mentions side-effects and so could include, 'What are the best ways to reduce, predict and manage the side-effects of treatment for children (including life threatening side-effects)?

Opting for questions focussed on intervention rather than description

Groups were clear that although it is useful to describe a problem, it is action through intervention that is required to improve children's and families' experiences. Therefore, 'Are the psychological, practical, and financial support needs of children with cancer, survivors, and their families being met during treatment and beyond? How can access to this support be improved and what further support would they like?' was placed higher in the rankings than 'What is the psychological and social impact of cancer and treatment on children and their families during treatment and in the long-term; what factors affect these impacts?' as the latter question involves description, rather than action.

Opting for questions that could have wider impact

Initially, most participants selected their top three questions reflective of their personal experience or area they worked within. During discussions, their opinions changed, and groups decided that the Top 10 questions should be generic and have the potential to have the greatest impact on as many children and families as possible. For example, 'How can experiences of having a Hickman line be improved for children with cancer?' was considered too specific and did not apply to all children.

Ensuring all themes within the questions were represented

Groups tried to cluster questions into similar themes, such as support, treatment, care, side-effects, their aim being to include each 'theme' in the Top 10. For example, the question about relapse was moved up during discussions as this was not covered by any other question.

Group discussion and decision-making

From the outset, there were some questions that were high priority for many and stayed high in the Top 10 throughout the workshop. The question ranked as top priority, 'Can we find effective and kinder (less burdensome, more tolerable, with fewer short and long-term effects) treatments for children with cancer, including relapsed cancer?' was the top priority for all three groups after the first group discussion. After the second group discussion, all three groups had the same questions ranked one to five, which remained in the same positions in the final Top 10.

The final group discussion focussed on whether to include, 'What is the relationship between chronic fatigue syndrome, fibromyalgia, chronic pain and treatment for childhood cancer?' in the Top 10 (it was at number 11). This push for inclusion came from two young adults who said these long-term effects had a huge impact on their lives and had experienced a lack of recognition and support. There was a group vote and the decision was made to move this question up to number 10 and move, 'What are the best ways to provide emotional support for children and their families 1) around the time of diagnosis, 2) during treatment and 3) after treatment (including survivors who are now adults)?' down to number 11 as this was covered by the broader question, about support at number 3.

The final Top 10 priorities are shown in Box 2 alongside the other 13 questions discussed.

For peer review only - http://bmjopen.bmj.com/site/about/guidelines.xhtml

For peer review only

For peer review only - http://bmjopen.bmj.com/site/about/guidelines.xhtml

Box 2 Top 10 research priorities for Children's cancer and the additional 13 questions discussed at the workshop

1. Can we find effective and kinder (less burdensome, more tolerable, with fewer short and long-term effects) treatments for children with cancer, including relapsed cancer?
2. Why do children develop cancer (including the role that genetics plays) and could it be prevented?[1]
3. Are the psychological, practical, and financial support needs of children with cancer, survivors, and their families being met during treatment and beyond? How can access to this support be improved and what further support would they like?[2]
4. How can we speed up the process of getting diagnosed and starting treatment in the right place?[1]
5. Why do children relapse, how can it be prevented, and what are the best ways to identify relapse earlier?
6. How can we make being in hospital a better experience for children and young people? (like having better food, internet, toys, and open visiting so other family members can be more involved in the child's care)[1]
7. What are the best ways to ensure children and families get and understand the information they need, in order to make informed decisions, around the time of diagnosis, during treatment, at the end of treatment and after treatment has finished?
8. What impact does cancer and treatment have on the lives of children and families after treatment, and in the long-term; what are the best ways to help them to overcome these impacts to thrive and not just survive?
9. How can we make more accessible treatments that are closer to home, in shared care hospitals?[1]
10. What is the relationship between chronic fatigue syndrome, fibromyalgia, chronic pain and treatment for childhood cancer? (Fibromyalgia is a long-term condition that causes pain all over the body.)

11. What are the best ways to provide emotional support for children and their families 1) around the time of diagnosis, 2) during treatment and 3) after treatment (including survivors who are now adults)?[1]
12. What are the best ways to reduce, predict and manage the side-effects of treatment for children (including life threatening side-effects)?
13. How can transition (moving) from child into adult services be improved for young people who had cancer as a child?
14. What is the psychological and social impact of cancer and treatment on children and their families during treatment and in the long-term; what factors affect these impacts?
15. How common are the different long-term effects of childhood cancer treatment, how do they change across the lifespan, can we predict them and how can they best be prevented, detected and/or treated?
16. What are the best ways to support the emotional wellbeing of professionals who care for children with cancer and their families?
17. During and after treatment, what issues prevent or encourage physical activity, which interventions are most effective and what should be measured to assess effectiveness?
18. What are the best ways of making sure people who had cancer as a child receive the information they need about the long-term effects of cancer and treatment?
19. What fertility preservation options work best for children and teenagers with cancer?
20. What are the long-term effects of additional medications children with cancer may receive (such as antibiotics, pain killers, laxatives) and how can these effects be reduced?
21. What are children's and survivors' experiences of the side-effects and long-term effects of cancer treatment?
22. How can experiences of having a Hickman line be improved for children with cancer? (A Hickman line is a small tube which is inserted into a vein so that treatments can be given, and blood taken without the repeated need to access veins with a needle. The Hickman line can stay in place for several months.)
23. What are the best ways to support children as they get older, and their needs change, to understand and take responsibility for their health, and to live with the long-term effects of cancer and treatment?

[1] These questions were in the Top 5 research priorities identified by children and young people.

[2] This question was originally not mapped onto the question about emotional support from children and young people, but the workshop participants decided that this question was related as it includes emotional support as well as other types of support.

For peer review only

For peer review only - http://bmjopen.bmj.com/site/about/guidelines.xhtml

DISCUSSION

The Children's Cancer PSP brought together children, survivors, families, and professionals to prioritise research questions on childhood cancer. The Top 10 priorities provide a resource to inform research funding decisions in government and charitable organisations. The top priority is, 'Can we find effective and kinder (less burdensome, more tolerable, with fewer short and long-term effects) treatments for children with cancer, including relapsed cancer?' This question was ranked as top in the shortlisting survey by all three respondent groups (patients/survivors, parents/relatives/friends, and professionals) and placed at number 1 from the start of workshop by all three discussion groups. This reflects shared priorities of continuing to improve cure rates whilst minimising treatment toxicity. The Top 10 priorities reflect the breadth of the cancer experience, including diagnosis, relapse, hospital experience, support during/after treatment and the long-term impact. Priorities highlight the need for research strategies to be holistic in their approach rather than solely driven by biological and drug intervention research. It is now critical that funders and researchers ensure future research focuses on what is important to children, survivors, families and professionals (9).

A number of cancer-related PSPs exist, including one in Canada also focusing on childhood cancer (https://www.jla.nihr.ac.uk/priority-setting-partnerships/pediatric-cancer/top-10-priorities.htm). The top priority for the Canadian PSP is preventing and managing treatment-related long-term effects which links to the top priority of our PSP and finding 'kinder' treatments. Both Top 10 lists feature similar questions on relapse, prevention/detection and questions about psychosocial impact and support. There is an increasing drive to focus on both physical and psychological health during and after cancer. It is already recognised that a cancer diagnosis has serious implications for children and young people's mental health during and after treatment (10,11), but this has yet to be systematically investigated, and how best to provide support remains unknown. Psychological support was the top priority in the Teenage and Young Adult Cancer PSP (12).

Challenges, strengths and limitations

The anticipated timeline for this project was two years, it took three. This delay was partly due to the Covid-19 pandemic. The project was resource intensive, requiring input from all steering group members. The challenge of involving professionals with full schedules, and parent/patient representatives with many concerns and commitments, was amplified by the pandemic, and our progress reflected this.

The scope of the PSP was intentionally broad to reflect the heterogeneity of childhood cancer, and variation in treatment and experience. This generated a significant workload when sorting and summarising diverse questions, and subsequent literature searching to verify uncertainties.

Engaging with children extended the project timescale; this work had to be carefully planned to ensure our methods were accessible and appropriate. Plans for face-to-face work were revised due to pandemic restrictions. Few priority setting exercises have involved many children and young people (6,13). Previous PSPs have reflected that they were unable to engage with children as they wished, due to lack of time and resources (14). It was of utmost importance to our steering group that children's voices were heard. We consider this aspect of our PSP a success: time and resources invested in engaging with children were worthwhile. Overall, questions from children reflected similar themes as those from adult participants, but there were some additional elements that featured as higher priority for children, such as having treatments closer to home and improving the hospital experience. In the final workshop, participants wanted children's voices to be heard, resulting in all five of the top priorities identified by children being reflected in the Top 10.

For peer review only - http://bmjopen.bmj.com/site/about/guidelines.xhtml

The use of the rigorous and transparent JLA methodology enhances the validity of the process and results. The response from parents/carers to both surveys was high and parent and patient representatives were involved in shaping the project from the outset, as members of the steering group. Their input was key; for example, they helped to ensure the surveys were presented in a user-friendly format and appropriate routes to dissemination were used. Parent/patient representatives reported a positive experience of being involved in the steering group*, "I wanted to be involved with the PSP because of the exciting opportunity to contribute towards future research topics in childhood cancer, bringing the voice of childhood cancer survivors from a service user perspective and advocating for the cohort. I have found the experience to be extremely positive and engaging. I feel that my presence is valued, and my contributions have been acknowledged and implemented throughout the process."*

Absent voices must be considered as a limitation. Of note, the majority of respondents described themselves as White. The priorities therefore represent the views of the majority, White population, which has been observed in other PSPs (15). Males were also underrepresented. We did not ask in the surveys whether respondents have a disability (whether resulting from treatment or not) and so cannot understand what impact this might have had on prioritisation.

Primary care has an important role in the care of children with cancer from diagnosis into survivorship (16). There was a primary care representative on the steering group and at the final workshop, but none responded to the initial survey, and only one to the shortlisting survey. The voices of these professionals are absent from the questions collected.

IMPLICATIONS AND DISSEMINATION

The Top 10 have been circulated on social media and via supporter newsletters/websites by the PSP funding charities and our Partners. Dissemination includes publication of a final report with an associated launch event, peer-reviewed publications, and conference presentations. We will report the detail of our engagement with children in a separate publication and are working with the JLA to develop guidance for future PSPs.

Our aspiration is that these prioritised questions will help to direct and shape future research. The uncertainties identified are the outcome of a systematic and transparent process and provide funders with clear guidance on the highest priorities for future research, voted on by end-users of research. Identifying clear areas for future research allows research funders to target funds effectively and inform fundraising activities. We plan to hold a meeting with funders to promote the priorities and encourage funding calls focused on the priority areas.

When selecting questions to be included in the Top 10, workshop participants intentionally opted for broad questions, to capture the widest range of issues. This is common in JLA PSPs, the questions therefore reflect broad topic areas for research; further refinement is required to transform topics into answerable research questions (17). This PSP also demonstrates that where sufficient expertise and resources are available, involvement of young children can be achieved. Therefore, funding guidance should encourage applicants to undertake such work.

Some questions submitted were outside the scope of the PSP and were removed. Many suggested a knowledge gap. The steering group considered these questions to be important and is determined to ensure these submissions are not 'lost'. We will look at how these questions, statements and service enquiries can be best used to improve information signposting. Questions were submitted regarding disparity in funding between childhood and 'adult' cancers. These questions were removed, as they are not amenable to research, but we intend to share them through a commentary piece, as they

For peer review only - http://bmjopen.bmj.com/site/about/guidelines.xhtml

reflected strong opinions and perceptions that would benefit from further exploration and articulation.

CONCLUSION

We have identified shared research priorities for children's cancer using a rigorous, person-centred approach involving stakeholders not typically involved in setting the research agenda, including children. Resulting questions reflect the breadth of the cancer experience for children and families, including diagnosis, relapse, hospital experience, support during and after treatment and the long-term impact of cancer. These must inform funding of future research, with priority questions evidenced by researchers.

AUTHOR CONTRIBUTIONS

All authors (SA, RH, BP, ABG, AB, JC, SC, RD, JG, NH, HH, JH, LH, LL, KM, SM, KMc, JM, HM, SP, SP, RRB, DS, AS, WTM, AW, AW, DW, FG) were part of the Children's Cancer Priority Setting Partnership steering group or coordinating team and made substantive contributions to the conduct of the study, overseeing all aspects of the work. All authors contributed to protocol design, survey refining, data cleaning and refining questions submitted in the initial survey. The project was managed by SA, FG (guarantor), BP, RH and JG. SA, FG, JM, SM, LL, KMc, RRB were part of a subgroup overseeing engagement with children throughout the PSP process. Specific contributions included: survey design (SA), coding the survey submissions (FG, SA), searching and checking uncertainties (SA, BP), managing data entry (SA). All authors reviewed and approved the final version of this paper.

ACKNOWLEDGEMENTS

The Children's Cancer PSP would like to thank everyone who took the time to send in their questions and vote on the importance of them. Thank you also to the children, young people, parents, relatives, and professionals who attended the workshops.

We would like to thank Angela Stewart for providing administrative support to the PSP. We would also like to thank the previous members of the steering group: Martin English, Penelope Hart-Spencer, Charmaine Jagger, and Angela Polanco.

FUNDING STATEMENT

This work was supported by Children's Cancer and Leukaemia Group (CCLG) and Little Princess Trust. No grant award number available.

Dr. Julia Chisholm is supported by the Giant Pledge through the Royal Marsden Cancer Charity and this independent research is supported by the National Institute for Health Research (NIHR) Biomedical Research Centre at The Royal Marsden NHS Foundation Trust and the Institute of Cancer Research, London. The views expressed are those of the authors and not necessarily those of the NIHR or the Department of Health and Social Care.

Professor Faith Gibson is supported in-part by the Great Ormond Street NIHR Biomedical Research Centre.

COMPETING INTERESTS

None declared.

PATIENT AND PUBLIC INVOLVEMENT

For peer review only

Patients and/or the public were involved in the design, or conduct, or reporting or dissemination plans of this research. See the Methods section for further details.

DATA SHARING STATEMENT

Further data regarding the original submissions to the surveys are available from: https://www.jla.nihr.ac.uk/priority-setting-partnerships/childrens-cancer/

ETHICAL APPROVAL

Ethical approvals are not required for JLA priority setting partnerships as per JLA and National Health Services Patient Safety Agency National Research Ethics Service guidance (https://www.invo.org.uk/posttypepublication/public-involvement-in-research-and-research-ethics-committee-review/).

REFERENCES

1. Cancer Research UK. Children's cancers incidence statistics 2018 [Available from: https://www.cancerresearchuk.org/health-professional/cancer-statistics/childrens-cancers/incidence accessed 20th April 2023.
2. Cancer Research UK. Children's cancers survival statistics 2016 [Available from: https://www.cancerresearchuk.org/health-professional/cancer-statistics/childrens-cancers/survival accessed 20th April 2023.
3. Chalmers I, Atkinson P, Fenton M, et al. Tackling treatment uncertainties together: the evolution of the James Lind Initiative, 2003-2013. *Journal of the Royal Society of Medicine* 2013;106(12):482-91. doi: 10.1177/0141076813493063
4. Crowe S, Fenton M, Hall M, et al. Patients', clinicians' and the research communities' priorities for treatment research: there is an important mismatch. *Research involvement and engagement* 2015;1:2. doi: 10.1186/s40900-015-0003-x
5. James Lind Alliance. The James Lind Alliance Guidebook. Version 10 2021 [Available from: https://www.jla.nihr.ac.uk/jla-guidebook/ accessed 13th April 2023.
6. Postma L, Luchtenberg ML, Verhagen AAE, et al. Involving children and young people in paediatric research priority setting: a narrative review. *BMJ Paediatr Open* 2022;6(1):e001610. doi: 10.1136/bmjpo-2022-001610
7. Aldiss S, Hollis R, Phillips B, et al. Research priorities in children's cancer. Final report of the James Lind Alliance Children's Cancer Priority Setting Partnership 2023 [Available from: https://www.jla.nihr.ac.uk/priority-setting-partnerships/childrens-cancer/ accessed 29th June 2023.
8. Aussems K, Schoemaker CG, Verwoerd A, et al. Research agenda setting with children with juvenile idiopathic arthritis: Lessons learned. *Child: care, health and development* 2022;48(1):68-79. doi: 10.1111/cch.12904
9. Gibson F, Aldiss S. What Are the Consequences of Not Responding to Research Priority Setting Exercises? *Cancer Care Research Online* 2023;3(1):e037. doi: 10.1097/cr9.0000000000000037
10. Berkman AM, Robert RS, Roth M, et al. A review of psychological symptoms and post-traumatic growth among adolescent and young adult survivors of childhood cancer. *J Health Psychol* 2022;27(4):990-1005. doi: 10.1177/1359105320971706
11. Sharkey CM, Espeleta HC, Traino KA, et al. Psychological adjustment outcomes among pediatric brain tumor survivors: A meta-analysis. *Pediatric blood & cancer* 2020;67(10):e28644. doi: 10.1002/pbc.28644
12. Aldiss S, Fern LA, Phillips RS, et al. Research priorities for young people with cancer: a UK priority setting partnership with the James Lind Alliance. *BMJ open* 2019;9(8):e028119. doi: 10.1136/bmjopen-2018-028119

For peer review only - http://bmjopen.bmj.com/site/about/guidelines.xhtml

For peer review only

13. Odgers HL, Tong A, Lopez-Vargas P, et al. Research priority setting in childhood chronic disease: a systematic review. *Archives of disease in childhood* 2018;103(10):942-51. doi: 10.1136/archdischild-2017-314631
14. Morris C, Simkiss D, Busk M, et al. Setting research priorities to improve the health of children and young people with neurodisability: a British Academy of Childhood Disability-James Lind Alliance Research Priority Setting Partnership. *BMJ open* 2015;5(1):e006233. doi: 10.1136/bmjopen-2014-006233
15. Drury NE, Herd CP, Biglino G, et al. Research priorities in children and adults with congenital heart disease: a James Lind Alliance Priority Setting Partnership. *Open Heart* 2022;9(2) doi: 10.1136/openhrt-2022-002147
16. Jain J, Qorri B, Szewczuk MR. The crucial role of primary care providers in the long-term follow-up of adult survivors of childhood cancer. *Cancer Manag Res* 2019;11:3411-18. doi: 10.2147/cmar.S197644
17. Staley K, Crowe S, Crocker JC, et al. What happens after James Lind Alliance Priority Setting Partnerships? A qualitative study of contexts, processes and impacts. *Research involvement and engagement* 2020;6:41. doi: 10.1186/s40900-020-00210-9

Figure 1 Pathway of care included in the project scope

Figure 2 Overview of the Children's Cancer Priority Setting Partnership methodology and results

For peer review only

For peer review only - http://bmjopen.bmj.com/site/about/guidelines.xhtml

Figure 1 Pathway of care included in the project scope

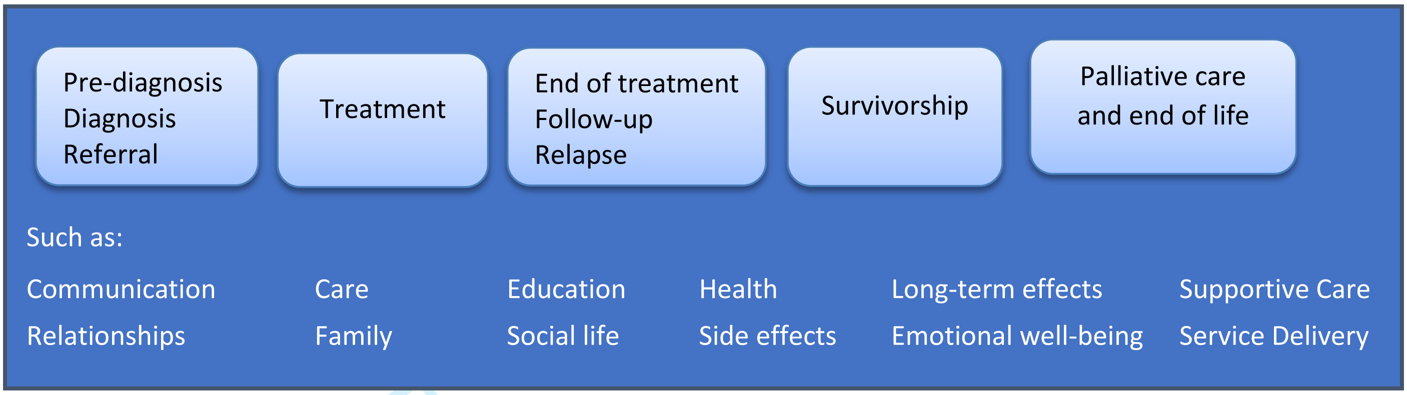

For peer review only

Figure 2 Overview of the Children's Cancer Priority Setting Partnership methodology and results

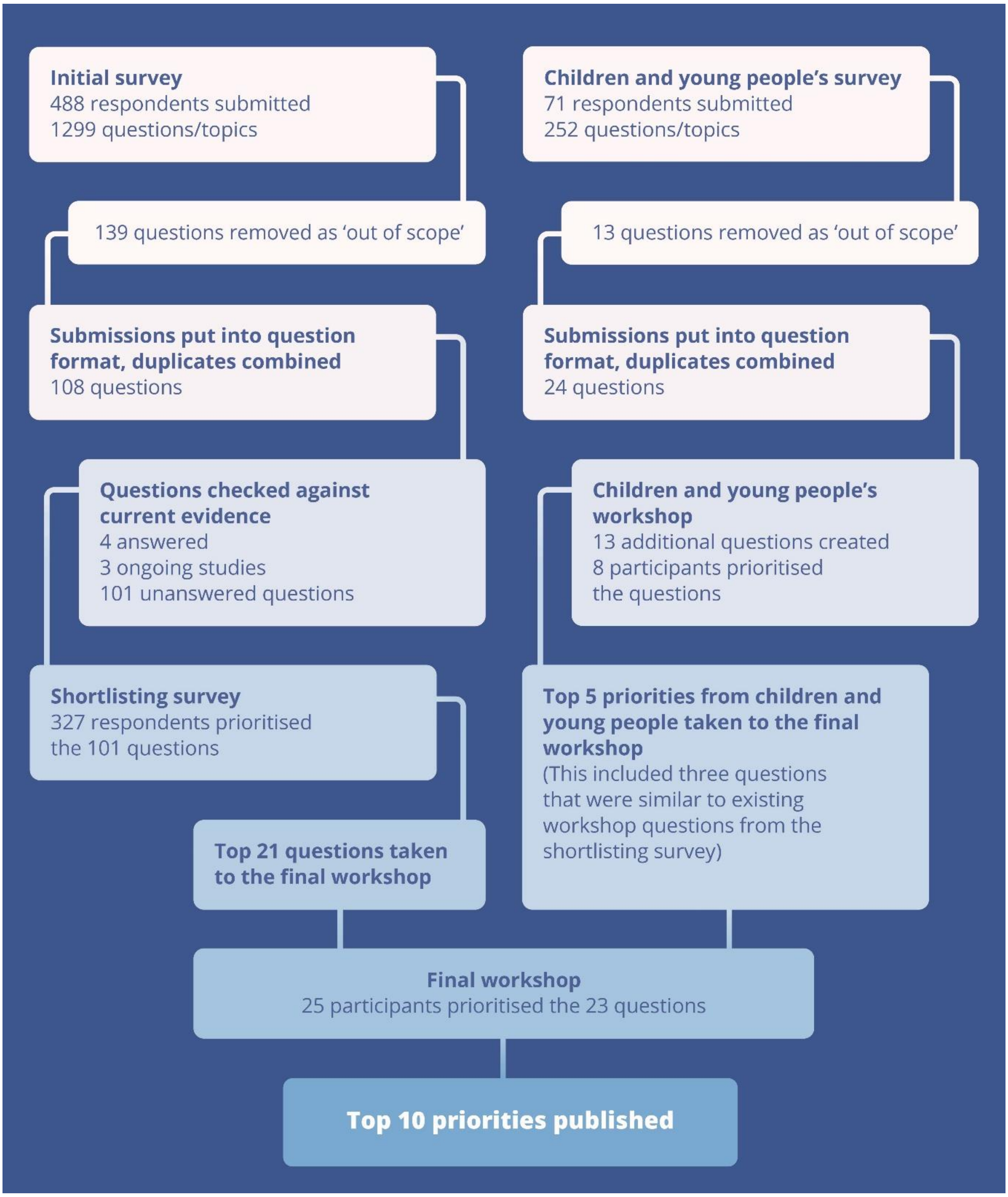

Supplementary material 1 Participant details first survey

	Response	Survivors (number=49)	Parents/relatives/friends/ (number=291)	Professionals (number=148)
**Gender**	Female	41 (84%)	260 (89%)	133 (90%)
	Male	8 (16%)	30 (10%)	14 (9%)
	Use another term	0 (0%)	0 (0%)	0 (0%)
	Prefer not to answer	0 (0%)	1 (0.3%)	1 (1%)
**Trans**	No	48 (98%)	281 (97%)	139 (94%)
	Yes	0 (0%)	0 (0%)	1 (1%)
	Prefer not to answer	0 (0%)	1 (0.3%)	0 (0%)
	Missing data	1 (2%)	9 (3%)	8 (5%)
**Ethnic group**	White	46 (94%)	276 (95%)	135 (91%)
	Asian or Asian British	1 (2%)	4 (1%)	6 (4%)
	Black African, Black Caribbean or Black British	0 (0%)	2 (1%)	2 (1%)
	Mixed/multiple ethnic groups	0 (0%)	4 (1%)	1 (1%)
	Other	2 (4%)	1 (0.3%)	2 (1%)
	Prefer not to answer	0 (0%)	2 (1%)	0 (0%)
	Missing data	0 (0%)	2 (1%)	2 (1%)
**Age (years)**	16-18	6 (12%)	0 (0%)	n/a
	19-24	4 (8%)	1 (0.3%)	3 (2%)
	25-34	18 (37%)	46 (16%)	33 (22%)
	35-44	12 (24%)	127 (44%)	39 (26%)
	45-54	7 (14%)	83 (29%)	46 (31%)
	55-64	1 (2%)	23 (8%)	23 (15%)
	65+	1 (2%)	5 (2%)	1 (1%)
	Prefer not to answer	0 (0%)	1 (0.3%)	1 (1%)
	Missing data	0 (0%)	5 (2%)	2 (1%)
**Country of residence (survivors/parents/relatives/friends) Country of work (professionals)**	England	36 (73%)	241 (83%)	123 (83%)
	Scotland	2 (4%)	12 (4%)	7 (5%)

For peer review only

	Wales	0 (0%)	13 (4%)	6 (4%)
	Northern Ireland	0 (0%)	4 (1%)	3 (2%)
	Other	10 (20%)	20 (7%)	8 (5%)
	Prefer not to answer	1 (2%)	1 (0.3%)	0 (0%)
	Missing data	0 (0%)	0 (0%)	1 (1%)
**Cancer first diagnosed with**	Bone tumour	6 (12%)	7 (2%)	n/a
	Brain or spinal tumour	3 (6%)	35 (12%)	n/a
	Germ cell tumour	0 (0%)	5 (2%)	n/a
	Kidney tumour	2 (4%)	18 (6%)	n/a
	Langerhans Cell Histiocytosis (LCH)	0 (0%)	5 (2%)	n/a
	Leukaemia	20 (41%)	132 (45%)	n/a
	Liver tumour	0 (0%)	1 (0.3%)	n/a
	Lymphoma	8 (16%)	19 (7%)	n/a
	Neuroblastoma	1 (2%)	22 (8%)	n/a
	Retinoblastoma	4 (8%)	14 (5%)	n/a
	Soft tissue sarcoma	4 (8%)	21 (7%)	n/a
	More than one cancer diagnosis	0 (0%)	5 (2%)	n/a
	Not sure	1 (2%)	2 (1%)	n/a
	Other	0 (0%)	5 (2%)	n/a
	Prefer not to answer	0 (0%)	0 (0%)	n/a
**Current situation**	On treatment	1 (2%)	90 (31%)	n/a
	Finished treatment in the last 0 to 12 months	0 (0%)	48 (16%)	n/a
	Finished treatment 1 to 5 years ago	3 (6%)	53 (18%)	n/a
	Finished treatment more than 5 years ago	40 (82%)	29 (10%)	n/a
	On treatment for relapse	2 (4%)	22 (8%)	n/a
	Receiving palliative care	0 (0%)	1 (0.3%)	n/a
	Passed away	n/a	45 (15%)	n/a
	Not sure	1 (2%)	0 (0%)	n/a
	Other	0 (0%)	3 (1%)	n/a
	Prefer not to answer	0 (0%)	0 (0%)	n/a
	Missing data	2 (4%)	0 (0%)	n/a

For peer review only

**Age at diagnosis**	Under 1	2 (4%)	24 (8%)	n/a
	1-3 years	9 (18%)	96 (33%)	n/a
	4-6 years	7 (14%)	66 (23%)	n/a
	7-9 years	9 (18%)	39 (13%)	n/a
	10-12 years	10 (20%)	26 (9%)	n/a
	13-15 years	11 (22%)	26 (9%)	n/a
	Over 16	0 (0%)	5 (2%)	n/a
	Not sure	0 (0%)	2 (1%)	n/a
	Prefer not to answer	0 (0%)	1 (0.3%)	n/a
	Missing data	1 (2%)	6 (2%)	n/a
**Professional group**	Allied health professional	n/a	n/a	49 (33%)
	Nurse	n/a	n/a	45 (30%)
	Doctor	n/a	n/a	27 (18%)
	Education professional	n/a	n/a	17 (11%)
	Social care professional	n/a	n/a	10 (7%)

For peer review only

Supplementary material 2 Participant details children and young people's surveys

	Response	Children and young people with cancer (n=61)	Siblings (n=10)
**Gender**	Male	22 (36%)	5 (50%)
	Female	38 (62%)	5 (50%)
	Prefer not to answer	1 (2%)	0 (0%)
**Age**	3-6 years	13 (21%)	1 (10%)
	7-9 years	17 (28%)	2 (20%)
	10-12 years	9 (15%)	2 (20%)
	13-15 years	16 (26%)	3 (30%)
	16-21 years	5 (8%)	1 (10%)
	Prefer not to answer	1 (2%)	1 (10%)
**Country of residence**	England	42 (69%)	6 (60%)
	Scotland	9 (15%)	2 (20%)
	Wales	6 (10%)	2 (20%)
	Northern Ireland	1 (2%)	0 (0%)
	Other	2 (3%)	0 (0%)
	Prefer not to answer	1 (2%)	0 (0%)
**Diagnosis**	Leukaemia	26 (43%)	3 (30%)
	Kidney tumour	7 (11%)	0 (0%)
	Lymphoma	7 (11%)	1 (10%)
	Brain/spinal tumour	5 (8%)	2 (20%)
	Soft tissue sarcoma	4 (7%)	0 (0%)
	Neuroblastoma	3 (5%)	2 (2%)
	Retinoblastoma	2 (3%)	0 (0%)
	Bone tumour	1 (2%)	0 (0%)
	More than one cancer diagnosis	1 (2%)	0 (0%)
	Other	2 (3%)	1 (10%)
	Prefer not to answer	2 (3%)	0 (0%)
	Do not know	1 (2%)	1 (10%)
**Ethnic group* (Children and young people with cancer n=36; Siblings n=7)**	White	31 (86%)	7 (100%)
	Asian or Asian British	1 (3%)	0 (0%)
	Black African, Black Caribbean or Black British	1 (3%)	0 (0%)
	Mixed/multiple ethnic groups	1 (3%)	0 (0%)
	Prefer not to answer	2 (6%)	0 (0%)
**Current situation* (Children and young people with cancer n=36; Siblings n=7)**	On treatment	12 (33%)	3 (43%)
	Finished treatment	23 (64%)	4 (57%)
	Other	1 (3%)	0 (0%)

*not asked in 4-7 year olds survey

For peer review only

Supplementary material 3 Participant details shortlisting survey

	Response	Survivors (number=27)	Parents/relatives/friends/ (number=210)	Professionals (number=90)
**Gender**	Female	23 (85%)	186 (89%)	75 (83%)
	Male	3 (11%)	21 (10%)	14 (16%)
	Use another term	1 (4%)	2 (1%)	0 (0%)
	Prefer not to answer	0 (0%)	0 (0%)	1 (1%)
	Missing data	0 (0%)	1 (0.5%)	0 (0%)
**Trans**	No	26 (96%)	206 (98%)	87 (97%)
	Yes	1 (4%)	0 (0%)	1 (1%)
	Prefer not to answer	0 (0%)	2 (1%)	2 (2%)
	Missing data	0 (0%)	2 (1%)	0 (5%)
**Ethnic group**	White	24 (89%)	199 (95%)	79 (88%)
	Asian or Asian British	1 (4%)	3 (1%)	5 (6%)
	Black African, Black Caribbean or Black British	0 (0%)	0 (0%)	1 (1%)
	Mixed/multiple ethnic groups	1 (4%)	5 (2%)	4 (4%)
	Other	1 (4%)	1 (0.5%)	0 (0%)
	Prefer not to answer	0 (0%)	1 (0.5%)	1 (1%)
	Missing data	0 (0%)	1 (0.5%)	0 (0%)
**Age (years)**	16-18	5 (19%)	1 (0.5%)	0 (0%)
	19-24	9 (33%)	3 (1%)	0 (0%)
	25-34	6 (22%)	24 (11%)	23 (26%)
	35-44	3 (11%)	98 (47%)	26 (29%)
	45-54	3 (11%)	57 (27%)	30 (33%)
	55-64	1 (4%)	19 (9%)	8 (9%)
	65+	0 (0%)	7 (3%)	1 (1%)
	Prefer not to answer	0 (0%)	0 (0%)	2 (2%)
	Missing data	0 (0%)	1 (0.5%)	0 (0%)
**Country of residence (survivors/parents/relatives/friends) Country of work (professionals)**	England	25 (93%)	170 (81%)	78 (87%)

For peer review only

	Scotland	0 (0%)	17 (8%)	6 (7%)
	Wales	0 (0%)	10 (5%)	3 (3%)
	Northern Ireland	0 (0%)	2 (1%)	1 (1%)
	Other	1 (4%)	10 (5%)	1 (1%)
	Prefer not to answer	1 (4%)	1 (0.5%)	1 (1%)
	Missing data	0 (0%)	0 (0%)	0 (0%)
**Cancer first diagnosed with**	Bone tumour	4 (15%)	6 (3%)	n/a
	Brain or spinal tumour	2 (7%)	26 (12%)	n/a
	Germ cell tumour	0 (0%)	3 (1%)	n/a
	Kidney tumour	0 (0%)	10 (5%)	n/a
	Langerhans Cell Histiocytosis (LCH)	0 (0%)	0 (0%)	n/a
	Leukaemia	10 (37%)	113 (54%)	n/a
	Liver tumour	0 (0%)	0 (0%)	n/a
	Lymphoma	4 (15%)	15 (7%)	n/a
	Neuroblastoma	1 (4%)	9 (4%)	n/a
	Retinoblastoma	1 (4%)	8 (4%)	n/a
	Soft tissue sarcoma	3 (11%)	14 (7%)	n/a
	More than one cancer diagnosis	1 (4%)	1 (0.5%)	n/a
	Not sure	0 (0%)	0 (0%)	n/a
	Other	1 (4%)	4 (2%)	n/a
	Prefer not to answer	0 (0%)	0 (0%)	n/a
	Missing data	0 (0%)	1 (0.5%)	n/a
**Current situation**	On treatment	2 (7%)	58 (28%)	n/a
	Finished treatment in the last 0 to 12 months	1 (4%)	21 (10%)	n/a
	Finished treatment 1 to 5 years ago	5 (19%)	60 (29%)	n/a
	Finished treatment more than 5 years ago	19 (70%)	30 (14%)	n/a
	On treatment for relapse	0 (0%)	8 (4%)	n/a
	Receiving palliative care	0 (0%)	2 (1%)	n/a
	Passed away	n/a	26 (12%)	n/a
	Not sure	0 (0%)	0 (0%)	n/a
	Other	0 (0%)	4 (2%)	n/a

For peer review only

	Prefer not to answer	0 (0%)	0 (0%)	n/a
	Missing data	0 (0%)	1 (0.5%)	n/a
**Age at diagnosis**	Under 1	1 (4%)	16 (8%)	n/a
	1-3 years	1 (4%)	63 (30%)	n/a
	4-6 years	6 (22%)	54 (26%)	n/a
	7-9 years	4 (15%)	25 (12%)	n/a
	10-12 years	3 (11%)	26 (12%)	n/a
	13-15 years	10 (37%)	23 (11%)	n/a
	Over 16	2 (7%)	1 (0.5%)	n/a
	Not sure	0 (0%)	0 (0%)	n/a
	Prefer not to answer	0 (0%)	0 (0%)	n/a
	Missing data	0 (0%)	2 (1%)	n/a
**Professional group**	Allied health professional	n/a	n/a	25 (28%)
	Nurse	n/a	n/a	30 (33%)
	Doctor	n/a	n/a	25 (28%)
	Education professional	n/a	n/a	4 (4%)
	Social care professional	n/a	n/a	4 (4%)
	Other	n/a	n/a	2 (2%)

For peer review only

Manuscript title: Research priorities for children's cancer: a James Lind Alliance priority setting partnership

Reporting guideline for priority setting of health research (REPRISE) - checklist

Tong A, Synnot A, Crowe S, Hill S, Matus A, Scholes-Robertson N, Oliver S, Cowan K, Nasser M, Bhaumik S, Gutman T, Baumgart A, Craig JC. Reporting guideline for priority setting of health research (REPRISE). BMC Med Res Methodol. 2019 Dec 28;19(1):243. doi: 10.1186/s12874-019-0889-3.

	**REPRISE reporting item**	**REPRISE descriptor and/or examples**	**Reported on Page #/in section**
**A**	**Context and scope**		
1	Define geographical scope	Global, regional, national, city, local area, institutional/organizational level, health service	Title, abstract and Introduction
2	Define health area, field, focus	Disease or condition specific, interventions, healthcare delivery, health system	Methods: Scope
3	Define the intended beneficiaries	This may include the general population or a specific population based on demographic (age, gender), clinical (disease, condition), or other characteristics who may benefit from the research	Introduction/discussion
4	Define the target audience of the priorities	Policy makers, funders, researchers, industry or others who have the potential to implement the priorities identified	Introduction/discussion
5	Identify the research area	Public health, health services research, clinical research, basic science	Methods: Scope
6	Identify the type of research questions	Etiology, diagnosis, prevention, treatment (interventions), prognosis, health services, psychosocial, behavioral and social science, economic evaluation, implementation; this may not be pre-defined	Methods: Scope and process
7	Define the time frame	Interim, short-term, long-term priorities, plans to revise and update	Not reported as no current plans to update, this will partly depend on whether questions are answered in future.
**B**	**Governance and team**		

8	Describe the selection and structure of the leadership and management team	Those responsible for initiating, developing, and guiding the process for priority setting, and examples of structures include; Steering Committee, Advisory Group, Technical Experts	Methods: Set-up
9	Describe the characteristics of the team	Stakeholder group or role, institutional affiliations, country or region, demographics (e.g. age sex), discipline, experience, expertise	Methods: Set-up
10	Describe any training or experience relevant to conducting priority setting	Consultants or advisers, members with experience or skills relevant to the conducting priority-setting e.g. qualitative methods, surveys, facilitation	Methods: Set-up
**C**	**Framework for priority setting**		
11	State the framework used (if any)	James Lind Alliance, COHRED, CHNRI, Dialogue Model, no framework (general research priority setting)	Methods
**D**	**Stakeholders or participants**		
12	Define the inclusion criteria for stakeholders involved in priority-setting	Patients, caregivers, general community, health professionals, researchers, policy makers, non-governmental organizations, government, industry; specific groups including vulnerable and marginalized populations	Methods: Process
13	State the strategy or method for identifying and engaging stakeholders	Partnership with organizations, social media, recruitment through hospitals	Methods: Process
14	Indicate the number of participants and/or organizations involved	Number of individuals and organizations, include number by stakeholder group	Results
15	Describe the characteristics of stakeholders	Stakeholder group, demographic characteristics, areas of interest and expertise, discipline, affiliations	Results and supplementary material 1,2 and 3
16	State if reimbursement for participation was provided	Cash, vouchers, certificates, acknowledgement; what purpose e.g. travel, accommodation, honorarium	PPI section
**E**	**Identification and collection of research priorities**		
17	Describe methods for collecting initial priorities	Methods e.g. Delphi survey, surveys, nominal group technique, interviews, focus groups, meetings, workshops; prioritization e.g. voting, ranking; mode e.g. face-to-face, online;	Methods: Process

		may be informed by evidence e.g. systematic reviews, reviews of guidelines/other documents, health technology assessment	
18	Describe methods for collating and categorizing priorities	Taxonomy or other framework used to organize, summarise, and aggregate topics or questions	Methods: Process
19	Describe methods and reasons for modifying (removing, adding, reframing) priorities	Based on scope, clarity, definition, duplication, other criteria	Methods: Process
20	Describe methods for refining or translating priorities into research topics or questions	Reviewed by Steering Committee or project team	Methods: Process
21	Describe methods for checking whether research questions or topics have been answered	Systematic reviews, evidence mapping, consultation with experts	Methods: Process
22	Describe number of research questions or topics	Number of priorities at each stage of the process	Methods: Results
**F**	**Prioritization of research topics/questions**		
23	Describe methods and criteria for prioritizing research topics or questions	Methods e.g. Delphi survey, surveys, nominal group technique, interviews, focus groups, meetings, workshops;<br>Prioritization e.g. voting, ranking;<br>Mode e.g. face-to-face, online;<br>Criteria e.g. need, feasibility, novelty, equity	Methods: Process
24	State the method or threshold for excluding research topics/questions	Thresholds for ranking scores, proportions, votes; other criteria	Methods: Process
**G**	**Output**		

25	State the approach to formulating the research priorities	Area, topic, questions, PICO (population, intervention, comparator, outcome)	Methods: Process
**H**	**Evaluation and feedback**		
26	Describe how the process of prioritization was evaluated	Survey, workshop	Results of JLA feedback surveys from the steering group and workshop are not reported due to word limits.<br>Feedback from a patient representative on steering group is included in the discussion section.
27	Describe how priorities were fed back to stakeholders and/or to the public; and how feedback (if received) was addressed and integrated	Public meetings or workshop, newsletters, website, email, online presentations	Implications and dissemination.
**I**	**Implementation**		
28	Outline the strategy or action plans for implementing priorities	Communication with target audience, via policies and funding	IMPLICATIONS AND DISSEMINATION
29	Describe plans, strategies, or suggestions to evaluate impact	Integration in decision-making, funding allocation, review of relevant documents<br><br>Again, this may be something that Steering Groups build into their earlier discussions about dissemination and implementation.	The James Lind Alliance try to track impact following PSPs (e.g. funding calls relating to PSP priorities). We have not reported this due to word limits.

J	**Funding and conflict of interest**		
30	State sources of funding	Name sources of funding for the priority-setting exercise; if relevant include the budget and/or cost	Funding statement
31	Declare any conflicts or competing interests	State any conflicts of interest that may be at an individual level and/or at a contextual level (e.g. political issues, controversies) that may affect the process, output or implementation.	Competing interests statement

For peer review only
